# Glycopolymers Mediate Suicide Gene Therapy in ASGPR-Expressing Hepatocellular Carcinoma Cells in Tandem with Docetaxel

**DOI:** 10.1021/acs.biomac.2c01329

**Published:** 2023-02-13

**Authors:** Daniela Santo, Rosemeyre A. Cordeiro, Patrícia V. Mendonça, Arménio C. Serra, Jorge F. J. Coelho, Henrique Faneca

**Affiliations:** † Center for Neuroscience and Cell Biology, University of Coimbra, Coimbra 3004-504, Portugal; ‡ Institute for Interdisciplinary Research, University of Coimbra, Coimbra 3030-789, Portugal; § Centre for Mechanical Engineering, Materials and Processes, Department of Chemical Engineering, University of Coimbra, Coimbra 3030-790, Portugal; ∥ Associação para a Inovação e Desenvolvimento Em Ciência e Tecnologia, IPN—Instituto Pedro Nunes, Rua Pedro Nunes, 3030-199 Coimbra, Portugal

## Abstract

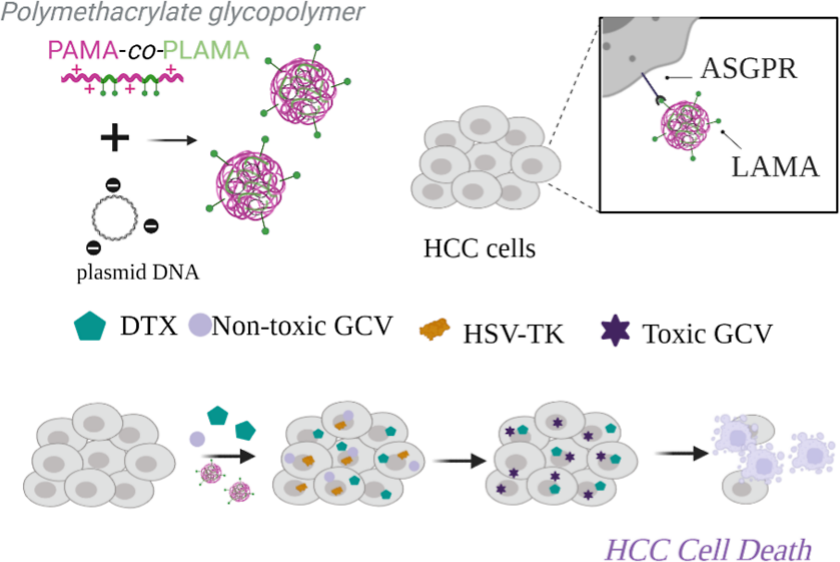

Cationic glycopolymers stand out as gene delivery nanosystems due to their inherent biocompatibility and high binding affinity to the asialoglycoprotein receptor (ASGPR), a target receptor overexpressed in hepatocellular carcinoma (HCC) cells. However, their synthesis procedure remains laborious and complex, with problems of solubilization and the need for protection/deprotection steps. Here, a mini-library of well-defined poly(2-aminoethyl methacrylate hydrochloride-*co*-poly(2-lactobionamidoethyl methacrylate) (PAMA-*co*-PLAMA) glycopolymers was synthesized by activators regenerated by electron transfer (ARGET) ATRP to develop an efficient gene delivery nanosystem. The glycoplexes generated had suitable physicochemical properties and showed high ASGPR specificity and high transfection efficiency. Moreover, the HSV-TK/GCV suicide gene therapy strategy, mediated by PAMA_144_-*co*-PLAMA_19_-based nanocarriers, resulted in high antitumor activity in 2D and 3D culture models of HCC, which was significantly enhanced by the combination with small amounts of docetaxel. Overall, our results demonstrated the potential of primary-amine polymethacrylate-containing-glycopolymers as HCC-targeted suicide gene delivery nanosystems and highlight the importance of combined strategies for HCC treatment.

## Introduction

Hepatocellular carcinoma (HCC) represents approximately 75–85% of primary liver cancers and is third most common cause of cancer death worldwide.^1^ In recent years, multiple kinase and immune checkpoint inhibitors have been approved as therapy approaches for patients with late-stage HCC.^2,3^ However, due to their restricted indications, low response rate, drug toxicity/resistance, and subsequent tumor relapse, the development of new antitumor strategies that can provide improved efficacy and reduced unexpected side effects is imperative.^4^

In this regard, gene therapy has become a promising therapeutic tool for cancer treatment.^5^ Particularly, suicide gene therapy represents approximately 6.1% (194 out of 3180) of all gene therapy clinical trials performed worldwide in 2021.^6^ The herpes simplex virus thymidine kinase gene (HSV-TK) is the most commonly used suicide gene and involves the concomitant treatment with the antiviral prodrug ganciclovir (GCV).^7,8^ This nontoxic prodrug is converted into activated toxic metabolites by the action of HSV-TK followed by endogenous kinases, causing cell death either by inducing chain termination during DNA synthesis or by affecting cell cycle progression.^9^ Unfortunately, the clinical translation of suicide gene therapy is still limited by the poor therapeutic outcome and the low cancer cell specificity.^10^ Therefore, the development of a high-performance suicide gene therapy strategy for the treatment of HCC requires the development of highly efficient and targeted gene delivery nanocarriers.

In this regard, sugar-based nanocarriers have been considered an appealing approach for the delivery of genetic material due to their inherent biocompatibility, colloidal stability, and tissue-specific targeting.^11,12^ The development of reversibly deactivated radical polymerization techniques, namely, atom transfer radical polymerization (ATRP) and reversible addition-fragmentation chain transfer polymerization (RAFT), allowed the synthesis of well-defined glycopolymers with precise molecular weight, diverse end-group functionalities, and different compositions, and a variety of architectures could be readily prepared.^13,14^ Lactobionic acid-functionalized nanocarriers, which are recognized by their biocompatibility and selective binding affinity, stand out as platforms for liver-specific gene delivery.^15^ These multifunctional galactosylated molecules display high binding affinity with the asialoglycoprotein receptor (ASGPR), an endocytic cell surface receptor overexpressed on liver cancer cells compared to hepatocytes,^16,17^ which make lactobionic-based nanocarriers a powerful tool to deliver therapeutic genes into tumor cells to combat HCC. Reineke and Narain’s groups have conducted a remarkable development on the research study of cationic glycopolymers and evaluation of their transfection ability as a function of various synthetic parameters, such as molecular weight,^18^ cationic content,^19,20^ carbohydrate content,^21^ and glycopolymer composition (random/block copolymers).^11,13−15^ Methacrylamide-based copolymers synthetized by copolymerization between primary amine monomers, such as 3-aminopropyl methacrylamide^22^ or 2-amino ethyl methacrylamide,^23,24^ and carbohydrate-derived monomers, namely, 3-gluconamidopropyl methacrylamide^25^ and 2-lactobionamidoethyl methacrylamide,^26,27^ are the most commonly used glycopolymers. Recently, Bockman et al. reported the synthesis of a *N*-acetyl-*d*-galactosamine (GalNAc)-derived monomer through a novel improved two-step route with high yield to prepare different diblock copolymers with 2-amino ethyl methacrylamide via RAFT polymerization. The transfection efficiency of the nanocarriers prepared with these glycopolymers was evaluated in HepG2 cells and depends on the GalNAc block length, which increases with the degree of polymerization (DP) of the carbohydrate moiety.^28^ In an another study, to understand the role of charge type of glycopolymers on the transfection efficiency, Haibo Li and co-workers synthetized various poly(2-deoxy-2-methacrylamido glucopyranose)-*b*-poly(methacrylate amine) block copolymers, bearing primary, secondary, tertiary, or quaternary amine functionality.^29^ Their results indicated that secondary-amine polymethacrylate-based copolymers exhibited higher gene delivery efficiency and lower cytotoxicity than glycopolymers containing more highly substituted amines. However, the potential of primary-amine polymethacrylate-containing glycopolymers as gene delivery nanosystems was not evaluated as this polymer was not hydrosoluble, a crucial characteristic of polymers for biomedical applications.^30^

**Scheme 1:**
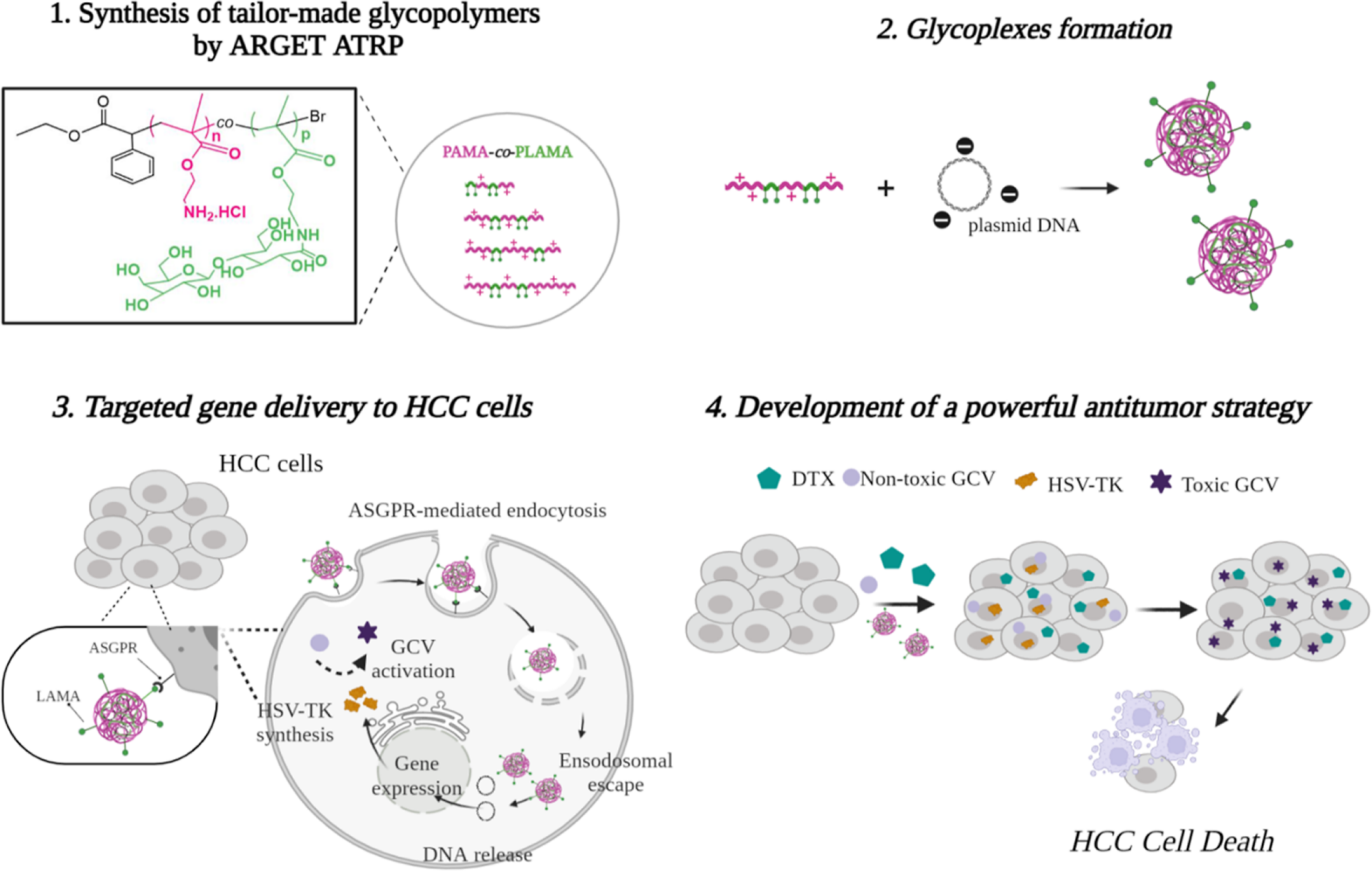
Schematic Illustration of the Proposed Anti-HCC Therapeutic Strategy Based on the Combinatorial Effects of DTX with Suicide Gene Therapy Mediated by PAMA-*co*-PLAMA-Based Glycoplexes

In this work, we proposed the development of a novel HCC-targeted glycopolymer-based nanocarrier to mediate suicide gene therapy, with powerful antitumor effect against HCC cells (Scheme 1). First, a mini-library of well-defined primary-amine polymethacrylate-based glycopolymers, with fixed DP of 2-lactobionamidoethyl methacrylate (LAMA) and different DP values of 2-aminoethyl methacrylate (AMA), was synthesized by activators regenerated by electron transfer (ARGET) ATRP. The effect of the primary amine content on the physicochemical properties, biological activity, biocompatibility, and ASGPR specificity of the nanocarriers were investigated. To boost the transfection efficiency and, thus the therapeutic potential of our best PAMA-*co*-PLAMA-based nanocarriers, HCC cells were pre-treated with a low concentration of docetaxel (DTX), a chemotherapeutic drug belonging to the category of microtubule depolymerization inhibitors.^31^ This drug binds to the β-subunit of the tubulin protein of the microtubules and promotes the hyperstabilization of microtubule assemblies, which impairs the mitotic progression and, consequently, leads to cell cycle arrest. Furthermore, as microtubules play a critical role in intracellular dynamics transport, including the trafficking of nanocarriers to lysosomes after their uptake by endocytosis, this drug may also be used to improve the transfection ability of nanocarriers by decreasing the entrapment inside the endosomal/lysosomal compartments.^32^ Therefore, our hypothesis was that DTX at low concentration could enhance the antitumor effect of HSV-TK/GCV suicide gene therapy, by increasing the transfection efficiency of glycoplexes and inhibiting mitosis. To the best of our knowledge, this was the first research study that combines DTX with suicide gene therapy mediated by methacrylate-based glycoplexes to combat HCC.

## Materials and Methods

### Materials

Information regarding materials is described in the Supporting Information.

#### Synthesis and Characterization of Glycopolymers

Details about techniques and equipment are described in the Supporting Information.

#### Typical Procedure for the Synthesis of PAMA-*co*-PLAMA by ARGET ATRP

AMA (1.51 g, 9.6 mmol), LAMA (0.6 g, 1.3 mmol), copper(II) bromide (CuBr_2_) (7.13 mg, 32 μmol), tris(pyridine-2-ylmethyl)amine (TPMA) (37.1 mg, 127 μmol), and ethyl α-bromophenyl acetate (EBPA) (15.5 mg, 64 μmol) were dissolved in the water/dimethylformamide (DMF) mixture (50/50, V/V) (3.5 mL). The mixture was purged with nitrogen for 30 min in the Schlenk flask. Then, the flask was placed under magnetic stirring at 60 °C, and a previously deoxygenated ascorbic acid (AscA) solution (43 mM) was continuously added to the mixture via a syringe pump at 1 μL/min during 4 h. The final reaction mixture was analyzed by ^1^H nuclear magnetic resonance (NMR) spectroscopy and by aqueous size exclusion chromatography (SEC). After that, it was dialyzed (dialysis membrane MWCO = 3500) against deionized water, and the glycopolymer was collected after the freeze-drying process.

#### Formulation of Polyplexes

Polymers were dissolved in ultra-pure water at pH 3 and blended with 1 μg of DNA plasmids encoding luciferase (pLuc), green fluorescent protein (pGFP), or HSV-TK (pTK) at the desired polymer/DNA N/P (+/−) charge ratio. The charge of the polymer corresponds to the DP of the monomers (each monomer unit has a positive charge at acidic pH). The mixture solution was incubated for 15 min and was immediately used.

#### Physicochemical Characterization of Polyplexes

The physicochemical properties of nanocarriers, namely, their ability to condense and protect DNA, their size, and their surface charge were assessed according to Santo et al., and the procedures are described in the Supporting Information.^38^

#### Transfection Activity and Interaction with Target Cells

The transfection activity, transfection efficiency, cell viability, and intracellular trafficking of the developed nanocarriers were evaluated according to Santo et al., and the used methodologies are reported in the Supporting Information.^38^

#### Antitumor Activity

The in vitro antitumor effect induced by non-viral HSV-TK/GCV gene therapy, DTX, or their combination was assessed in 2D and 3D HepG2 cell culture models. The procedures used for 3D cell cultures are described in the Supporting Information.

For a 2D cell culture model, following 4 h incubation of HepG2 cells with PAMA_144_-*co*-PLAMA_19_-based polyplexes, in the presence or absence of different concentrations of DTX (0.006; 0.003; 0.0125; 0.0250; 0.5; and 0.1 μM), the cell culture medium was renewed with DMEM-HG containing 10% (V/V) FBS. 24 h after, the cell culture medium was renewed with DMEM-HG with or without 100 μM of GCV, and cells were further incubated for 5 days in 5% CO_2_ at 37 °C. The cell viability was evaluated at 24, 72, and 120 h by Alamar Blue assay. After each measurement, the cell culture medium (with or without GCV) was renewed. In addition, the cell viability was also assessed at 120 h through sulforhodamine B (SRB) assay.^33^

The cell death mechanisms were assessed by flow cytometry using FITC-Annexin V and propidium iodide (PI) probes in 24-well culture plates. After 72 h of incubation with PAMA_144_-*co*-PLAMA_19_/pTK; PAMA_144_-*co*-PLAMA_19_/pTK + GCV; DTX; PAMA_144_-*co*-PLAMA_19_/pTK + DTX; and PAMA_144_-*co*-PLAMA_19_/pTK + GCV + DTX, cells were harvested, washed, and resuspended in 100 μL of binding buffer [10 mM HEPES (pH 7.4), 2.5 mM CaCl_2_, and 140 mM NaCl] containing 2 μL of FITC-annexin V and 1 μL of PI (0.05 mg/mL). Cells were incubated at RT and protected from light for 5 min and then analyzed (10,000 events) in a FACS Calibur flow cytometer (Becton Dickinson, USA). The data were analyzed using FlowJo software. Fluorescence images were obtained using fluorescein diacetate (5 mg/mL in acetone) and PI (2 mg/mL in PBS) for live/dead staining of HepG2 cells. After incubating for 15 min, the cells were washed and fixed with 4% paraformaldehyde solution (15 min, RT). The images were acquired with 20x magnification on an Axio Imager Z2 microscope (Zeiss, Munich, Germany) coupled to AxioCam HRc camera (Zeiss, Germany).

#### Statistical Analysis

All the results correspond to mean ± standard deviation, achieved from triplicates and are representative of at least three independent experiments. Data were analyzed by GraphPad Prism (version 6.01 GraphPad Software Inc., San Diego, CA, USA) using one-way analysis of variance (ANOVA) followed by the Dunnett test or using two-way ANOVA followed by Dunnett or Sidak tests. For all tests, statistical significance was considered for *p*-values <0.05.

## Results and Discussion

### Synthesis and Characterization of PAMA-co-PLAMA Glycopolymers

**Scheme 2:**
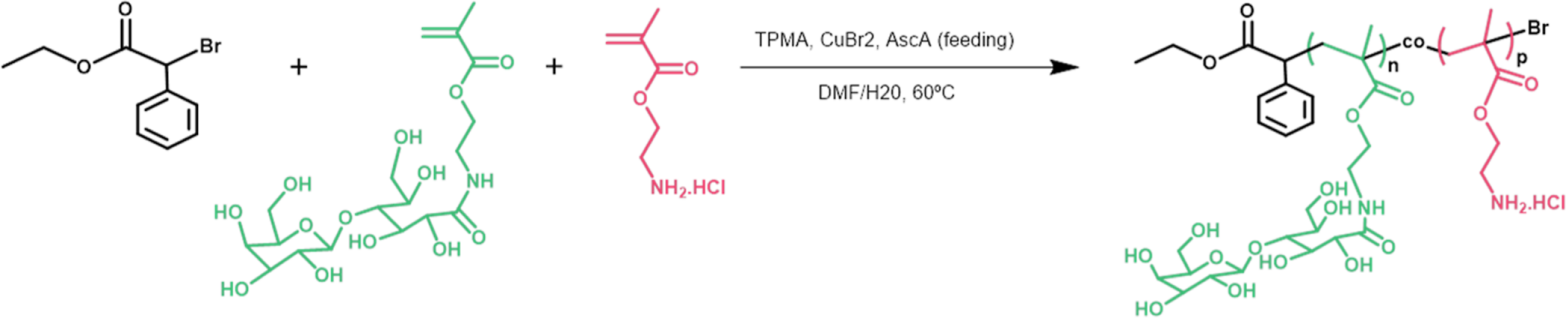
Synthesis of the Random PAMA-*co*-PLAMA Glycopolymers by ARGET ATRP

To prepare new methacrylate-based glycopolymers with well-defined, functionalized, and controlled structures which further developed a highly efficient and hepatocyte-specific gene delivery nanocarrier, a series of random PAMA-*co*-PLAMA glycopolymers were synthetized by ARGET ATRP. Lactobionic acid displays high binding affinity with the ASGPR and is capable of forming an amide bond between its carboxyl group and the amine groups of monomers or functional polymers, making lactobionic acid an ideal molecule for selectively targeting HCC cells.^17,34^ In this regard, LAMA, an inexpensive lactobionic-acid derivative monomer, was synthetized by reacting AMA with lactobionolactone, without protecting group chemistry.^35^ The chemical structure of the LAMA monomer was confirmed by ^1^H and ^13^C NMR spectroscopies (Figure S1, Supporting Information), and it is in agreement with data reported in the literature.^35^ Then, LAMA and AMA, a primary amine-containing monomer that will ensure the polyplex formation via electrostatic interaction with genetic material, were further polymerized through ARGET ATRP. Armes’ group reported the polymerization of LAMA and AMA, by ATRP in 3:2 methanol/water mixtures or in isopropanol/water mixtures using the CuBr/bipyridine complex as the catalyst, to obtain different AMA- and LAMA-block copolymers.^35,36^ However, for biomedical applications, the synthesis of glycopolymers by ARGET ATRP is advantageous as it allows control over the molecular weight of the polymers using a much lower concentration of the metal catalyst.^37^ Here, the synthesis of well-defined random copolymers was performed using a slow feeding of AscA as a reducing agent in the 1:1 DMF/water mixture at 60 °C, without protecting group chemistry, avoiding the typically troublesome multistep protection/deprotection reactions of glycopolymers synthesis (Scheme 2).

The chain length of LAMA was fixed (DP = 20) and different DP values of AMA (DP = 55, 73, 88, and 144) were targeted to evaluate the effect of the cationic content on the physicochemical properties, transfection capacity, cytotoxicity, and targeting ability of the methacrylate-containing glycopolymers-based gene delivery nanocarriers (Table 1).

**Table 1:** Composition and Molecular-Weight Parameters of Glycopolymers Prepared by ARGET ATRP^a^

	DP				
polymer sample	AMA	LAMA	*M*_n_^th^ × 10^3^^b^	*M*_n_^SEC^ × 10^3^^c^	*Đ*
PLAMA_38_		38	18.4	25.4	1.05
PAMA_161_^d^	161	N/A	25.7	26.9	1.10
PEG_45_-*b*-PAMA_168_^d^	168	N/A	29.9	28.8	1.10
PAMA_55_-*co*-PLAMA_21_	55	21	19.4	18.9	1.37
PAMA_73_-*co*-PLAMA_21_	73	21	22.0	23.4	1.36
PAMA_88_-*co*-PLAMA_20_	88	20	24.0	27.6	1.32
PAMA_144_-*co*-PLAMA_19_	144	19	33.0	38.7	1.31

a *M*_n_, number-average molecular weight; *D̵*, dispersity (*M*_w_/*M*_n_).

b Determined from monomer conversion. *M*_n_^th^ = [(AMA conversion/100) × DP_AMA_ × M_w,AMA_] + [(LAMA conversion/100) × DP_LAMA_ × M_w,LAMA_] + M_w,EBPA_.

c Determined by SEC using conventional calibration with PEG standards.

d The synthesis and characterization of PAMA_161_ and PEG_45_-*b*-PAMA_168_ were previously reported.^38^

A PAMA_161_ homopolymer and a PEG_45_-*b*-PAMA_168_ block copolymer were also synthesized through the same technique, to be used as control samples, as we previously confirmed the potential of these polymers as gene delivery nanocarriers.^38^ All polymers were purified by dialysis against water and collected by the freeze–drying process, yielding solids with high water solubility, which enabled their application in gene delivery. Then, their chemical structure was analyzed by ^1^H NMR spectroscopy and SEC (Figures S2 and S4 Supporting Information).

The results showed that the developed ARGET ATRP method yielded homopolymers and random glycopolymers with good control over molecular weight (*Đ* < 1.4), as illustrated by representative monomodal SEC chromatograms (Figure S3, Supporting Information) (Table 1).

The synthesis of these copolymers with controlled structure is very important to clarify the relationship between chemical structures and biological properties.

### Transfection Activity and Cytotoxicity of Glycopolymer-Based Polyplexes

**Figure 1:**
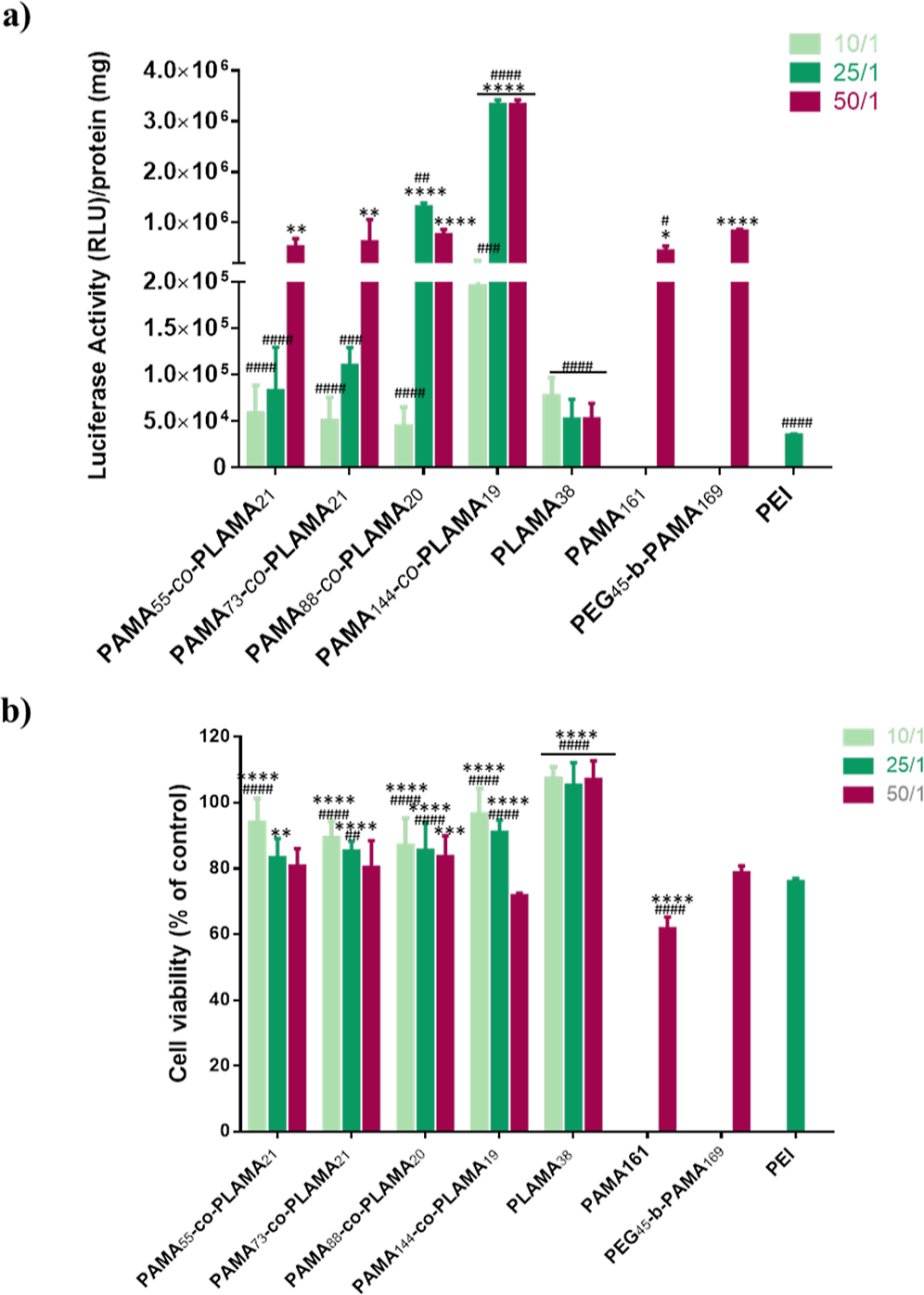
Transfection activity (a) and cytotoxicity (b) of PAMA-*co*-PLAMA-based nanocarriers in HepG2 cells. Polyplexes were prepared by complexing glycopolymers, PAMA_161_ and PEG_45_-*b*-PAMA_168_ with DNA plasmid encoding luciferase at different N/P ratios. Asterisks (****p < 0.0001, ***p < 0.001, **p < 0.01, and *p < 0.05) and cardinals (####*p* < 0.0001, ###*p* < 0.001, and #*p* < 0.05) indicate values with statistical significance when compared to those obtained with the standard formulations, PEI-and PEG_45_-*b*-PAMA_168_-based polyplexes, respectively.

To the best of our knowledge, the effect of the primary amine content on the transfection activity and cytotoxicity of methacrylate-based glycoplexes in gene delivery has never been studied. Therefore, to evaluate the potential of PAMA-*co*-PLAMA glycopolymers as gene delivery nanosystems, a preliminary study was performed in HepG2 cells using luciferase as a reporter gene (Figure 1).

As shown in Figure 1a, the ability of the different nanocarriers to effectively deliver plasmid DNA into HepG2 cells depends on their N/P ratio and composition. The transfection activity of the developed PAMA-*co*-PLAMA-based polyplexes was generally improved by the increase of the tested N/P ratios. Nanocarriers prepared with PAMA_55_-*co*-PLAMA_21_ and PAMA_73_-*co*-PLAMA_21_ copolymers presented the highest gene reporter expression for 50/1 N/P ratios, while nanovehicles prepared with PAMA_88_-*co*-PLAMA_20-_ and PAMA_144_-*co*-PLAMA_19_ glycopolymers, which have higher content of AMA, exhibited high levels of biological activity even for 25/1 N/P ratios. This fact could be related to higher amounts of cationic polymers, which establish multiple electrostatic interactions with endosomal membranes, facilitating the escape of genetic material from the endolysosomal pathway to the cytoplasm.^39^ In addition, the results showed that the carbohydrate homopolymer PLAMA_38_-based nanosystems promoted low transgene expression. This poor performance as gene delivery nanocarriers can be explained by their low ability to condense the genetic material, consequently allowing the premature release of DNA and/or enabling its degradation before reaching the nucleus (Figure S6d, Supporting Information). Moreover, the results demonstrate that PAMA_144_-*co*-PLAMA_19_-based nanosystems presented the highest biological activity, exhibiting higher transgene expression than that PAMA_161_- and PEG-*b*-PAMA_168_-based polyplexes. This is a remarkable result because it has been reported that the glycopolymer-based nanosystems usually present lower transfection efficiency than the corresponding cationic homopolymer-based nanocarriers.^15,22^

Additionally, all the developed formulations exhibited higher transfection activity than that obtained with the gold-standard polymer for gene delivery application—the polyethylenimine (PEI). Cytotoxicity is one of the most common shortcoming of polymeric-based gene delivery nanosystems.^30^ To overcome this drawback, without affecting the gene delivery efficacy, some modifications of cationic polymers with different biocompatible molecules, such as PEG, have been explored.^38^ In this regard, functionalization of cationic polymers with carbohydrates, ubiquitous components of biological systems, has been considered an attractive strategy to improve the biocompatibility of polymeric-based nanosystems.^40^

As illustrated in Figure 1b, the viability of HepG2 cells depends on the N/P ratio of polyplexes and their composition. In general, PAMA-*co*-PLAMA-based nanocarriers induced low cytotoxicity, with cell viability exceeding 80% for all formulations, except for PAMA_144_-*co*-PLAMA_19_-based complexes, which were prepared at an N/P/ratio of 50/1 and exhibited a cytotoxicity of 30%. This reduction in cell viability can be explained by the multiple and strong interactions of the nanosystems with the cytomembranes, causing their destabilization and, consequently, influencing the metabolic activity of the cells.^41^ Nevertheless, the data obtained showed that polyplexes based on PAMA_144_-*co*-PLAMA_19_ prepared at a 25/1 N/P ratio presented an excellent biocompatible profile and induced much lower cytotoxicity than PAMA_161_-, PEG_45_-*b*-PAMA_168_, and PEI-based nanocarriers.

**Figure 2:**
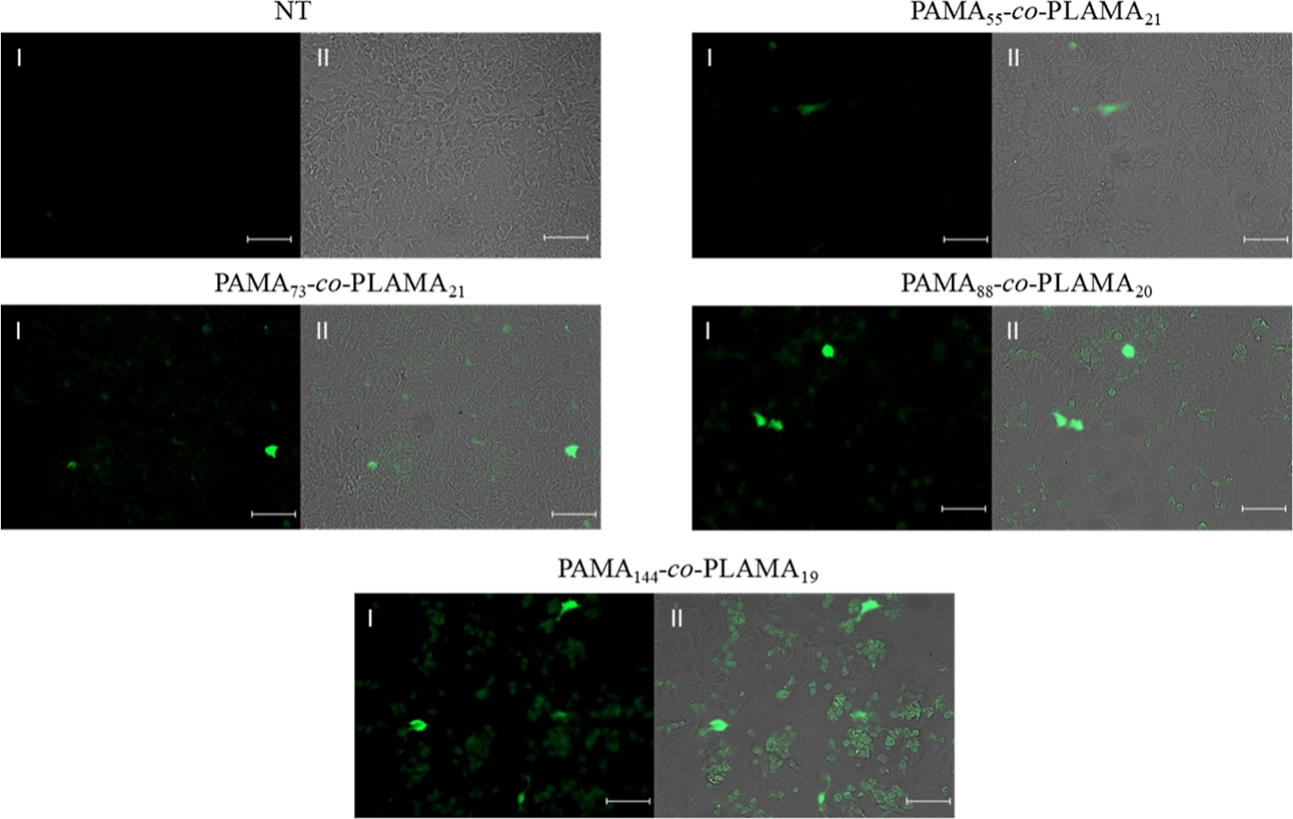
Transfection efficiency assessed by fluorescence microscopy in HepG2 cells. Typical fluorescence images (I) and overlapping (II) of fluorescence microscopy and phase contrast images of cells after transfection with different glycopolymer-based nanocarriers (scale bar = 50 μm).

To assess the influence of the AMA content on the transfection efficiency of PAMA-*co*-PLAMA-based nanocarriers, fluorescence microscopy was performed after transfection of HepG2 cells with glycoplexes prepared with plasmid DNA encoding green fluorescent protein (pGFP) (Figure 2).

The obtained results revealed that the transfection efficiency was improved by the increase of the polymerization of AMA, with the highest number of GFP-expressing cells being observed after transfection with PAMA_144_-*co*-PLAMA_19_-based polyplexes (Figure 2). In addition, these nanosystems promoted a large number of transfected cells than that obtained with PEG_45_-*b*-PAMA_168_- or PEI-based polyplexes (Figure S7, Supporting Information).

### Asialoglycoprotein Receptor-Targeted Nanocarriers

To improve the biocompatibility of cationic-based nanosystems, glycopolymers have been widely used as multivalent ligands to target lectin receptors overexpressed on the surface of cancer cells. The ASGPR binds specifically galactose moieties of desialylated glycoproteins, and the binding affinity augments with the valence of the carbohydrate residues, a phenomenon termed as the cluster glycoside effect.^42^ To determine whether the developed PAMA-*co*-PLAMA-based polyplexes are specifically recognized by the ASGPR of HCC cells, a competition assay of transfection in the presence of asialofetuin, a natural ligand of ASGPR, or an antibody against the ASGPR was performed.

**Figure 3:**
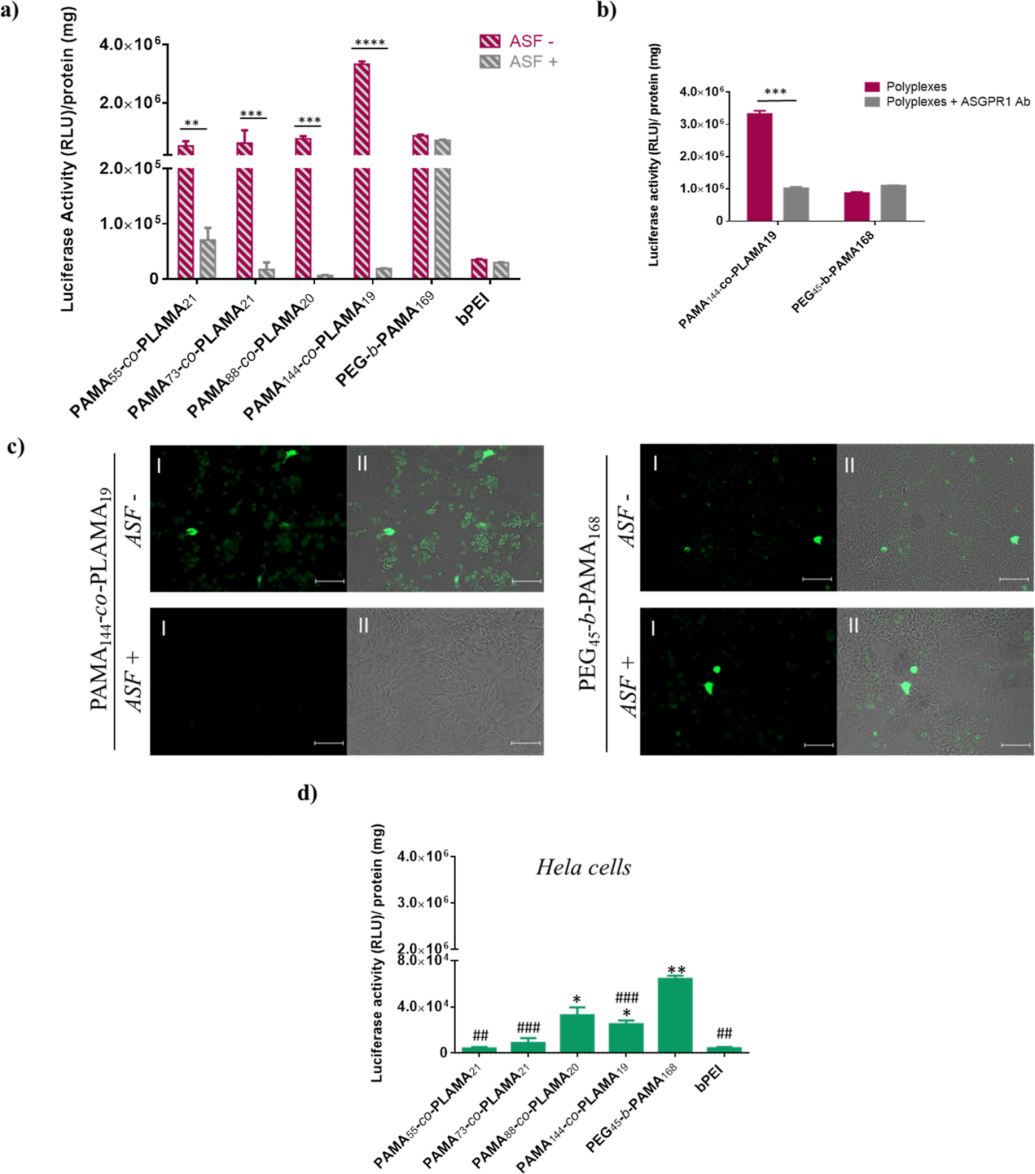
Effect of the presence of asialofetuin and an antibody against the ASGPR on biological activity of PAMA-*co*-PLAMA-based polyplexes in HepG2 cells. Biological activity of polyplexes in Hela cells. (a,b) Asterisks (****p < 0.0001, ***p < 0.001, **p < 0.01, and *p < 0.05) correspond to values that differ significantly from those obtained with the same formulations in the absence of asialofetuin or Ab against ASGPR. (c) Typical fluorescence images (I) and overlapping of fluorescence microscopy and phase contrast images (II) of cells transfected with different nanocarriers in the presence and absence of asialofetuin (scale bar = 100 μm). (d) Asterisks (**p < 0.01 and *p < 0.05) and cardinals (###*p* < 0.001 and ##*p* < 0.01) indicate values with statistical significant differences when compared to those obtained with the PEI-based nanosystem at a 25/1 N/P ratio, or with PEG_45_-*b*-PAMA_168_-based polyplexes, respectively.

The data presented in Figure 3a showed that the pretreatment of HepG2 cells with asialofetuin drastically reduced the transfection activity of PAMA-*co*-PLAMA-based polyplexes, while it did not significantly change the biological activity of PEI- and PEG_45_-*b*-PAMA_168_-based nanocarriers. In addition, the effect of the preincubation of HepG2 cells with an antibody against the ASGPR on the biological activity of PAMA_144_-*co*-PLAMA_19_-based nanocarriers revealed that this pretreatment induced a strong reduction in their transfection activity. However, it did not affect the biological activity of PEG_45_-*b*-PAMA_168_-based nanocarriers (Figure 3b). To further confirm that our most promising formulation, PAMA_144_-*co*-PLAMA_19_-based nanocarriers prepared at a 25/1 N/P ratio, was specifically recognized by the ASGPR of HepG2 cells, the effect of asialofetuin on the transfection efficiency was also evaluated by fluorescence microscopy (Figure 3c). The results confirmed that asialofetuin bound to the ASGPR blocks the internalization of the developed formulation, consequently resulting in a significantly lower number of GFP-expressing cells than that observed in the absence of asialofetuin. On the other hand, the data showed that PEG-*b*-PAMA_168_- or PEI-based nanocarriers promoted similar transfection efficiencies, regardless of the presence or absence of asialofetuin, which highlights the fact that the developed glycoplexes are specifically recognized by the ASGPR expressed on the surface of HepG2 cells (Figures 3c and S7). These assays were also performed in Hep3B cells, and a similar decrease in the transfection activity of the developed glycoplexes was observed in the presence of the ASGPR-competition agent, being this effect more pronounced with PAMA_144_-*co*-PLAMA_19_-based nanocarriers (Figure S8, Supporting Information). In addition, the biological activity of PAMA-*co*-PLAMA-based polyplexes was also investigated in cells not expressing ASGPR (Hela cells) (Figure 3d).^15^ The results showed that the higher transfection activity of PLAMA-containing nanocarriers obtained in HepG2 and Hep3B cells, compared with PEI-based nanosystems, was not observed in Hela cells. In fact, in these cells, the highest transfection efficiency was obtained with the non-targeted nanocarriers PEG-*b*-PAMA_168_-based polyplexes. Overall, these results demonstrated that the developed nanocarriers with galactose residues have the ability to specifically bind ASGPR, enhancing both their cell internalization and transfection activity.

### Endocytosis and Intracellular Fate of Glycoplexes

**Figure 4:**
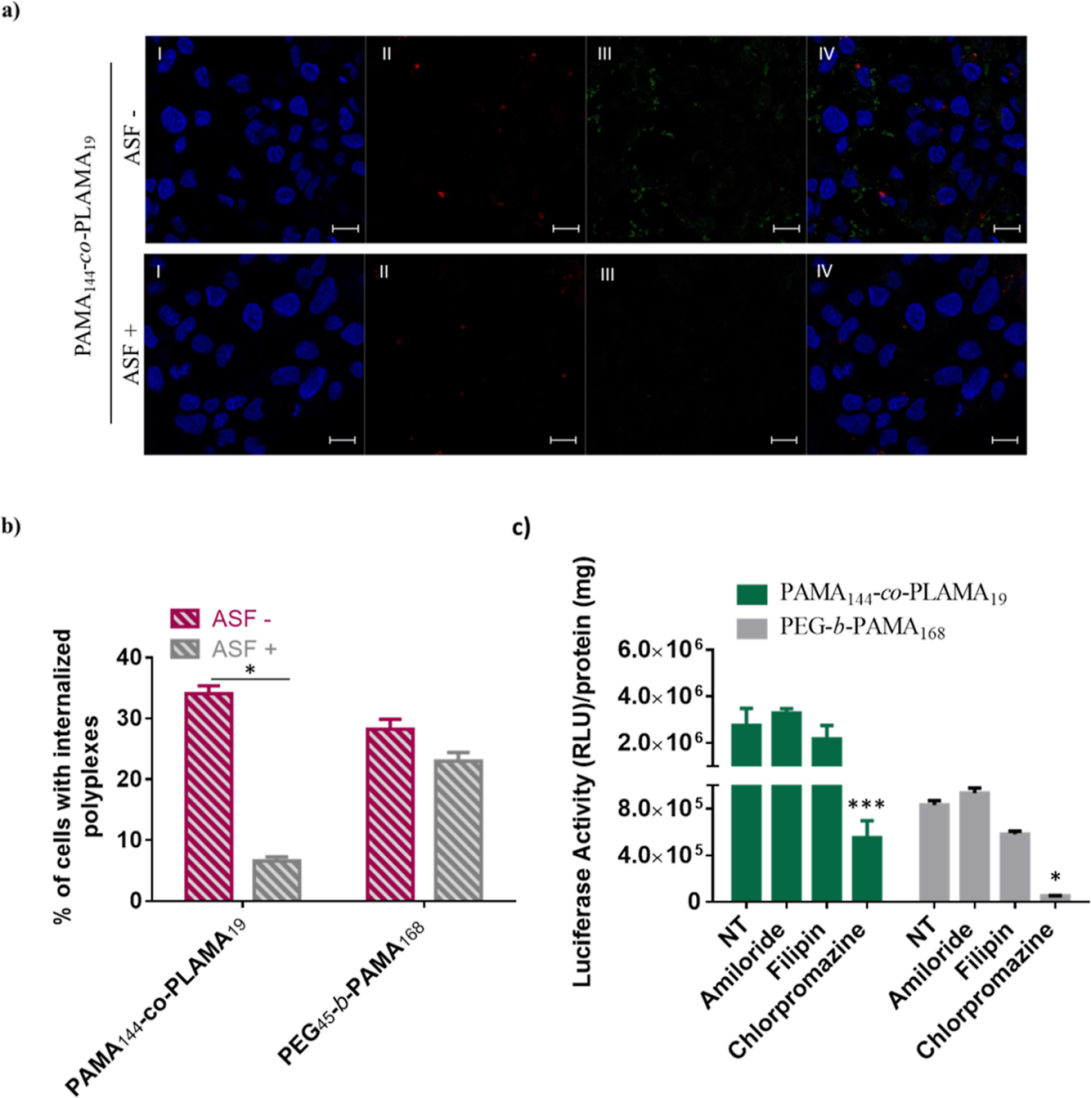
Effect of the presence of asialofetuin on the cellular uptake of PAMA_144_-*co*-PLAMA_19_ and PEG_45_-*b*-PAMA_168_-based polyplexes, evaluated by confocal microscopy (a) and flow cytometry (b) and the influence of endocytosis inhibitors on their transfection ability (c). (a) Representative confocal microscopic images of HepG2 cells treated with PAMA_144_-*co*-PLAMA_19_-based nanocarriers (scale bar = 10 μm): (I) cell nucleus stained by DAPI (blue); (II) acidic cellular compartments stained with Lysotracker Red DND-99 (red); (III) polyplexes prepared with 1% fluorescein-labeled glycopolymer (green); and (IV) overlay of images I–III. (b) Asterisks (*p < 0.05) correspond to values which differed significantly from those obtained with the same formulations in the absence of asialofetuin. (c) HepG2 cells were treated or not treated (Nt) with endocytosis inhibitors: chlorpromazine (50 mM), filipin (1 μg/mL), and amiloride (0.25 mM). Asterisks (***p < 0.001 and *p < 0.05) indicate values with statistical significance compared to those measured in untreated cells (control).

The transfection efficiency of non-viral vectors is conditioned by multiple intracellular barriers as the cellular uptake and endolysosomal escape to nuclear internalization.^43,44^ The interaction of nanocarriers with cells, and consequently their internalization, is affected by several parameters, in particular by their physicochemical properties and glycopolymer composition.^21^ The cellular internalization of the developed nanocarriers, containing 1% fluorescein-labeled PAMA-*co*-PLAMA glycopolymer, was evaluated by confocal microscopy and flow cytometry, in the presence or absence of asialofetuin (Figure 4ab).

The results, as shown in Figure 4a, indicated that cellular internalization of PAMA_144_-*co*-PLAMA_19_-polyplexes was significantly inhibited by pretreatment with asialofetuin. In addition, colocalization was not observed between the developed glycoplexes and lysosomal compartments (red fluorescence), which suggests that they have the ability to efficiently escape from the endolysosomal pathway to the cytoplasm, avoiding the subsequent DNA degradation in acidic cellular compartments. This observation can probably be explained by the high content of PAMA, a polymer containing primary amines that may interact and disrupt the endocytic membranes promoting the release of nanocarriers into the cell cytoplasm.^45^ Furthermore, ASGPR-mediated cellular internalization of the developed nanocarriers was confirmed by flow cytometry (Figure 4b). As expected, the cellular internalization of PAMA_144_-*co*-PLAMA_19_-based nanocarriers decreased significantly in the presence of asialofetuin, whereas the uptake of PEG_45_-*b*-PAMA_168_-based polyplexes was not affected by the pretreatment with this glycoprotein. This result can be explained by the binding of asialofetuin to the ASGPR, which blocks the internalization of the LAMA-containing nanosystems, but not the internalization of PEG_45_-*b*-PAMA_168_-based nanocarriers, since the latter do not interact with the ASGPR. The extension of the interaction between glycopolymers and specific lectins depends on their molecular weight, composition, arrangement in the nanoparticle structure, and length of spacer groups between polymer backbone and the pendant carbohydrate groups.^22,46^ For a fixed amount of carbohydrates, the increase of DP value of AMA resulted in a substantial decrease of the cellular internalization of PAMA-*co*-PLAMA-based nanocarriers (Figure S5, Supporting Information). Despite the higher cellular internalization of PAMA_55_-*co*-PLAMA_21_-based polyplexes when compared to PAMA_144_-*co*-PLAMA_19_-based ones, their transfection activity was much lower, suggesting the higher ability of our best formulation to effectively overcome the multiple intracellular barriers. The p*K*_a_ values of PAMA-*co*-PLAMA glycopolymers likely help explain these differences between nanocarriers with different DP of AMA (Figure S5, Supporting Information). The PAMA_144_-*co*-PLAMA_19_ glycopolymer has the lower p*K*_a_ value and, consequently, the lower degree of protonation at physiological pH, resulting in a higher buffering capacity. The endocytic pathway has a critical role in the intracellular trafficking of nanocarriers and, consequently, in the transfection efficiency.^47^ To evaluate the endocytic pathway involved in the cellular uptake of PAMA_144_-*co*-PLAMA_19_-based nanocarriers, their transfection activity was measured in the presence of various endocytic inhibitors (Figure 4c). Clathrin-mediated endocytosis was inhibited using chlorpromazine, caveolae-mediated endocytosis was blocked through the treatment with filipin, and amiloride was used to hinder macropinocytosis. Different concentrations of each endocytic inhibitor were used to define the lower drug concentration at which the inhibitor was efficient, without provoking significant toxicity (Figures S10 and S11, Supporting Information). In addition, the endocytic pathways engaged in the cellular uptake of non-targeted nanocarriers—PEG-*b*-PAMA_168_-based nanosystems—were also evaluated. As shown in Figure 4c, pretreatment of HepG2 cells with chlorpromazine resulted in a significant reduction on transgene expression, suggesting that the clathrin-coated pit endocytic pathway is involved in the uptake of the developed PAMA_144_-*co*-PLAMA_19_-based nanocarriers. The physicochemical properties of these glycoplexes, namely, their size of approximately 144 nm, were compatible with their internalization by clathrin-mediated endocytic pathway (Figure S6, Supporting Information). Moreover, this result confirmed the ASGPR-mediated cellular internalization, due to the specific binding of the galactose residues to this receptor. Regarding the PEG_45_-*b*-PAMA_168_-based polyplexes, the obtained data showed that the transgene expression was also negatively affected by the preincubation with chlorpromazine, indicating the involvement of clathrin-mediated endocytosis. This transfection activity reduction was not due to the toxicity of the endocytic inhibitors as no significant changes in cell viability were observed for the selected concentrations (Figures S10 and S11, Supporting Information).

### Antitumor Activity

DTX is a standard first-line chemotherapeutic drug for the treatment of various cancers. However, clinical trials indicate that DTX does not seem to be safe and effective enough for patients suffering from advanced HCCs.^48^ This anticancer agent has multiple target processes, including apoptotic, angiogenic, and gene expression ones.^31^ In addition, as an inhibitor of microtubule depolymerization, this second-generation taxane may also decrease the intracellular traffic of polyplexes to lysosomes, enhancing the transfection efficiency of non-viral vectors.^49,50^ The HSV-TK/GCV gene therapy strategy focus on the delivery into tumor cells of a gene encoding the enzyme HSV-TK that metabolizes GCV to GCV monophosphate, which in turn is phosphorylated to the triphosphate form by cellular kinases.^51^ Since the latter compound is an analogue of deoxyguanosine triphosphate, the inhibition of DNA polymerase and/or incorporation into DNA will occur, resulting in chain termination and tumor cell death.^52^ Moreover, suicide gene therapy yields better therapeutic outcomes due to the bystander effect involving the neighboring cancer cells, thus suppressing the necessity to deliver therapeutic genetic material to all tumor cells.^7^

Considering that one of the goals of this work was to develop an effective anti-HCC strategy, we investigated whether DTX as a transfection enhancer, together with its anticancer activity, would improve the therapeutic potential of the suicide gene therapy strategy mediated by the developed glycoplexes. To this end, we analyzed the effect of different DTX concentrations on luciferase gene expression in HepG2 cells transfected with PAMA_144_-*co*-PLAMA_19_/DNA nanosystems prepared at a 25/1 N/P ratio.

**Figure 5:**
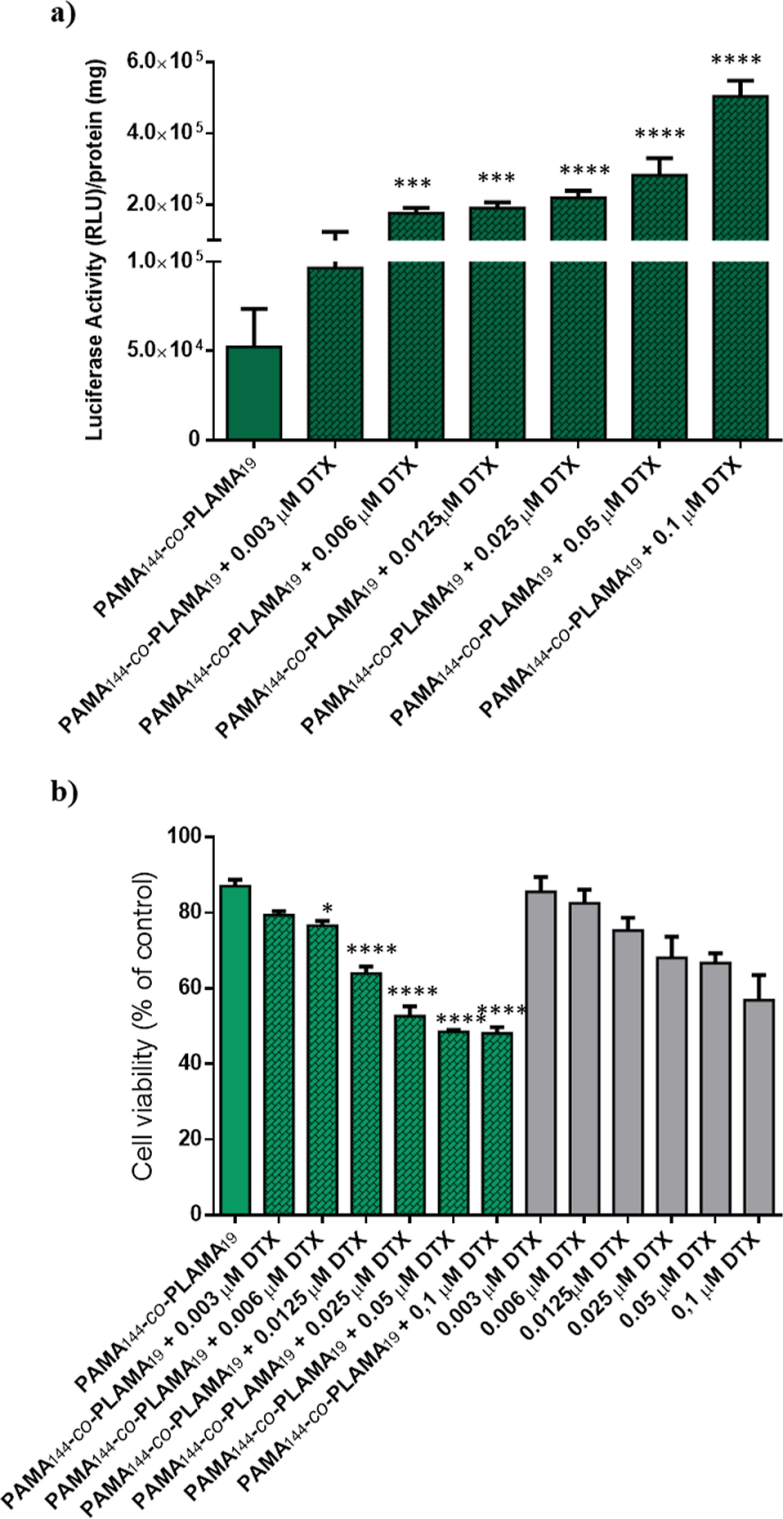
Effect of the DTX concentration on the biological activity (a) and cytotoxicity (b) of PAMA_144_-*co*-PLAMA_19_-based glycoplexes, prepared at a 25/1 N/P ratio, in HepG2 cells. Asterisks (****p < 0.0001, ***p < 0.001, **p < 0.01, and *p < 0.05) indicate values that significantly differ from those measured for PAMA_144_-*co*-PLAMA_19_-based nanocarriers in the absence of DTX.

The data presented in Figure 5a showed that pretreatment of HCC cells with DTX resulted in an increase of the biological activity of the nanocarriers. This booster effect was concentration-dependent and reached the highest effect at 0.1 μM DTX, under the conditions tested and limited by the drug cytotoxicity. The cytoskeleton plays a pivotal role in different cellular processes, namely, the maintenance of cell shape, mitosis, cell motility, and intracellular trafficking of nanoparticles. Filopodia, actin projections that extend from the cell surface, actively detect glycoplexes in the extracellular milieu and internalize these nanoparticles into clathrin-coated vesicles, which are then transported along microtubules to the main cell body to deliver nucleic acids to the nucleus.^47^ As DTX binds to the β-subunit of the tubulin protein of the microtubules and promotes the hyperstabilization of microtubule assemblies, it probably prevents the transport of glycoplexes to lysosomes. Thus, as previously reported by several authors, the increase in transgene expression induced by microtubule-targeting agents is probably due to the enhancement of intracellular trafficking from the endocytic pathway to the nucleus rather than an increase in cellular binding and internalization of nanosystems.^53,54^ Increasing the concentration of DTX resulted in high levels of cytotoxicity, and as DTX treatment was performed in the free form, it was critical to select a concentration that enhanced transfection activity without affecting the cell viability (Figure 5b). Therefore, the concentration of 0.006 μM, which tripled the transgene expression of the developed nanocarriers, was selected for further studies, namely those involving the delivery of the therapeutic gene.

In this regard, to evaluate the therapeutic potential resulting from the combination of the suicide gene therapy strategy with a low concentration of DTX, HepG2 cells were treated with free drug or with PAMA_144_-*co*-PLAMA_19_-based nanosystems carrying the pTK plasmid, in the presence (combined therapy) or absence of DTX, followed by incubation for 5 days with 100 μM of GCV.

**Figure 6:**
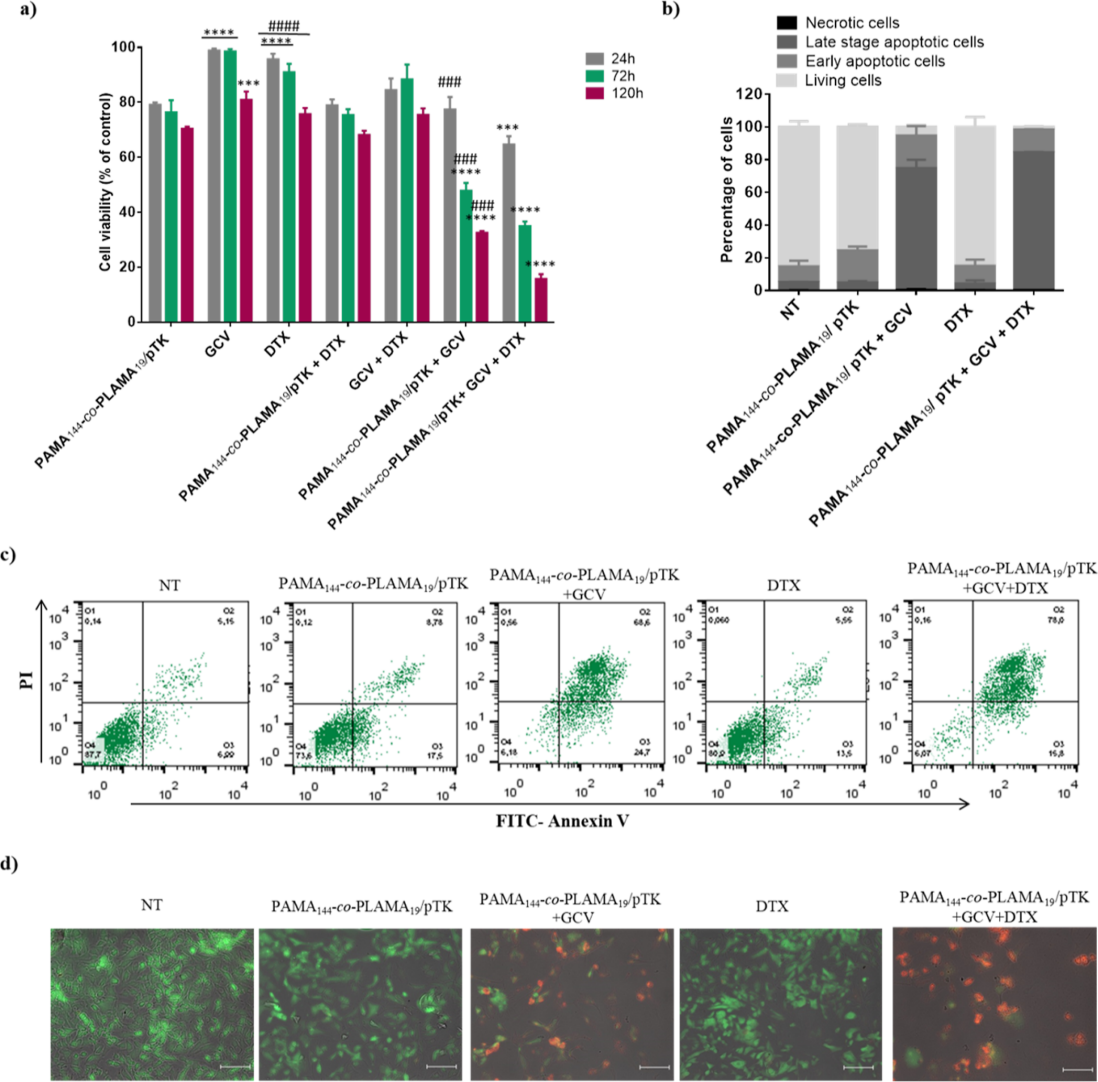
Therapeutic potential of the suicide gene therapy strategy mediated by the glycopolymer-based nanocarrier combined with DTX. Effect on viability (a) and apoptosis levels of HepG2 cells (b,–d). HepG2 cells were treated with different antitumor strategies: suicide gene therapy (PAMA_144_-*co*-PLAMA_19_/pTK + GCV), chemotherapy (free DTX), and suicide gene therapy combined with chemotherapy (PAMA_144_-*co*-PLAMA_19_/pTK + GCV + DTX). (a) Data are expressed as the percentage of cell viability with respect to untreated cells (control). Asterisks (****p < 0.0001 and ***p < 0.001) indicate values that significantly differ from those measured for cells transfected with PAMA_144_-*co*-PLAMA_19_-based nanocarriers, containing 1 μg of pTK plasmid. Cardinals (####*p* < 0.0001 and ###*p* < 0.001) correspond to data from cells treated with each individual strategy (PAMA_144_-*co*-PLAMA_19_/pTK + GCV or DTX) which significantly differ from those obtained with cells treated with the combined therapy (PAMA_144_-*co*-PLAMA_19_/pTK + GCV + DTX). (b) Percentage of viable, early apoptotic, late apoptotic/necrotic and necrotic cells obtained from flow cytometry analysis, measured after 72 h of treatment. (c) Representative scatter plots of FITC-annexin V vs PI. Q1, necrotic cells; Q2, late apoptotic/necrotic cells; Q3, early apoptotic cells; and Q4, viable cells. (d) Representative images of overlapping fluorescence microscopy and phase contrast of cells using fluorescein diacetate (green) and PI (red) staining for imaging live and dead cells, respectively (scale bar = 50 μm).

As shown in Figure 6, the cytotoxic effect promoted by the HSV-TK/GCV gene therapy strategy or the combined approach is time-dependent, being the highest cytotoxic effect observed at the fifth day of treatment. After transfection with PAMA_144_-*co*-PLAMA_19_/pTK-based nanocarriers, followed by 5 days of treatment with 100 μM GGV, 68% of cell death was achieved. On the other hand, the treatment of non-transfected cells with DTX resulted only in a slight decrease of cell viability. However, the preincubation of cells with 0.006 μM DTX, followed by 5 days of treatment with 100 μM GGV resulted in 85% of cytotoxicity, showing an additive effect promoted by the combination of these two therapeutic approaches. As shown in Figure 6a, GCV was not toxic to non-transfected cells, either per se or in the presence of DTX, and no significant toxicity was measured upon transfection of cells in the absence of GCV treatment. To confirm these data, cytotoxicity was also measured through SRB assay, which allows determination of cell viability in terms of cell proliferation, based on the protein content relatively to untreated control cells, instead of metabolic activity. The results showed that the combined therapeutic strategy indeed produced a higher level of toxicity than that obtained with the individual approaches (Figure S12 Supporting Information). This additive effect was probably a consequence of the multiple effects associated with the combined strategy. Most notably, the small amount of DTX, which by itself does not cause a significant toxicity, could enhance the effect of the gene therapy strategy, by binding to beta-tubulin, inhibiting microtubule depolymerization, and consequently enhancing gene expression HSV-TK. Moreover, DTX many not only improve gene therapy strategy but also directly contribute to the antitumor effect by arresting the cell cycle at the mitosis level and reducing the expression of anti-apoptotic genes.^31^

The molecular mechanism of cell death involved in the antitumor activity of the combined and individual strategies was evaluated by cell staining with annexin V and PI. As shown in Figures 6b,c, cells treated with our proposed combined therapeutic strategy (PAMA_144_-*co*-PLAMA_19_/pTK + GCV + DTX) had a higher percentage of non-viable apoptotic and/or necrotic cells after 72 h of incubation than cells treated with the individual strategies (PAMA_144_-*co*-PLAMA_19_/pTK + GCV or DTX). Furthermore, the negligible toxic effect of 0.006 μM DTX confirmed the hypothesis that this chemotherapeutic drug, at this concentration, merely enhances the transfection ability of the developed glycoplexes, thereby increasing the success of the suicide gene therapy strategy. Moreover, a large number of necrotic (red) than viable cells (green) were observed with the developed combined strategy, compared with the individual therapeutic strategies (Figures 6 and S12, Supporting Information).

3D tumor spheroids have been used to overcome 2D culture constraints, by providing more realistic spatial/structural architecture and biophysiological relevant information, bridging the experimental gap between in vivo and in vitro results.^55,56^ In order to evaluate the robustness of these therapeutic approaches, the antitumor effect of the combined strategy (PAMA_144_-*co*-PLAMA_19_/pTK + GCV + DTX) and of the individual approaches, suicide gene therapy (PAMA_144_-*co*-PLAMA_19_/pTK + GCV) and chemotherapy (DTX), was examined in HepG2 tumor spheroids.

**Figure 7:**
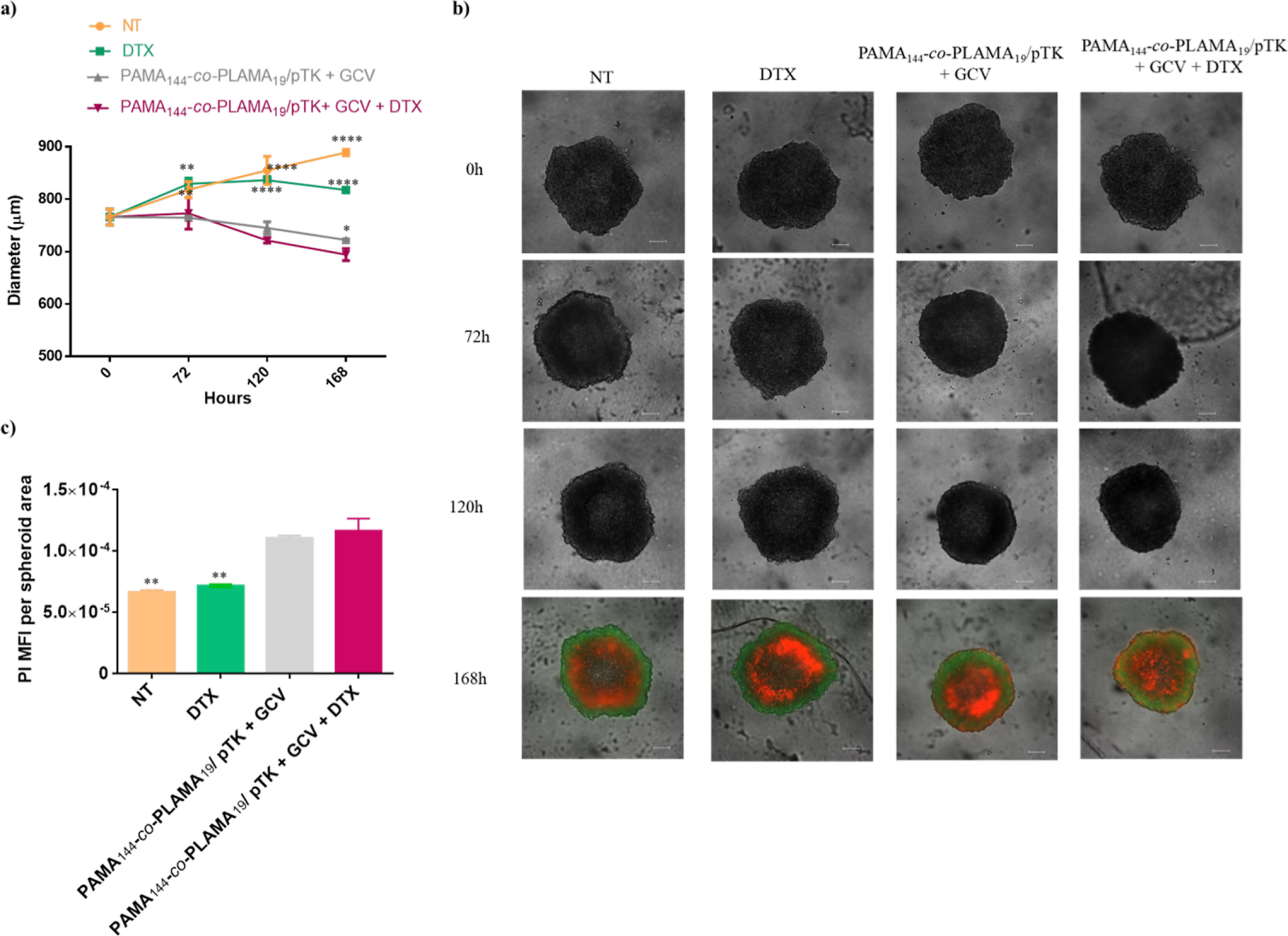
Effect of the suicide gene therapy strategy mediated by the glycopolymer-based nanocarriers combined with DTX on the tumor spheroid growth. HepG2-spheroids were treated with different antitumor strategies: suicide gene therapy (PAMA_144_-*co*-PLAMA_19_/pTK + GCV) and DTX and suicide gene therapy combined with DTX (PAMA_144_-*co*-PLAMA_19_/pTK + GCV + DTX). (a) Asterisks (****p < 0.0001, ***p < 0.001) correspond to data achieved with spheroids treated with each individual strategy and non-treated control, which significantly differ from those obtained with spheroids treated with the combined therapy (PAMA_144_-*co*-PLAMA_19_/pTK + GCV + DTX). (b) Microscopic images (scale bar = 200 μm) from 0 to 120 h are phase contrast images, and the microscopic images for 168 h are fluorescence images using fluorescein diacetate (green) and PI (red) staining for imaging live and dead cells, respectively. (c) PI mean fluorescence intensity (MFI) per spheroid area.

The results presented in Figure 7a,b show that after 168 h of transfection with PAMA_144_-*co*-PLAMA_19_/pTK-based nanocarriers, followed by treatment with 100 μM of GGV, the size of tumor spheroids decreased by 30%, whereas no significant change in their diameter was observed in non-transfected cells treated with 0.006 μM DTX. However, tumor spheroids treated with our proposed combined therapeutic strategy (PAMA_144_-*co*-PLAMA_19_/pTK + GCV + DTX) showed a reduction of 38% in the size. In addition, the PI mean fluorescence intensity (MFI) measurements showed that spheroids treated with PAMA_144_-*co*-PLAMA_19_/pTK + GCV or with PAMA_144_-*co*-PLAMA_19_/pTK + GCV + DTX exhibited a higher PI MFI per spheroid area than those treated with DTX or non-treated ones (Figure 7c), demonstrating the high therapeutic potential of suicide gene therapy combined with DTX.

## Conclusions

In summary, we developed a novel glycopolymer-based nanocarrier and evaluated the antitumor effect resulting from the combination of HSV-TK suicide gene therapy, mediated by these HCC-targeted nanosystems, with low concentrations of DTX. To this end, a series of water-soluble PAMA-*co*-PLAMA random glycopolymers were synthesized by ARGET ATRP (without protection/deprotection chemistry). These polymethacrylate-based glycopolymers have shown to be capable of forming nanosystems for gene delivery with suitable physicochemical properties, high transfection efficiency, biocompatibility, and ASGPR specificity. In addition, our best formulation, PAMA_144_-*co*-PLAMA_19_-based polyplexes, showed excellent performance as suicide gene therapy mediators, with substantial antitumor effects enhanced by combination with DTX, even in hard-to-transfect multicellular tumor spheroids. Overall, the obtained results show the great potential of the PAMA_144_-*co*-PLAMA_19_ glycopolymer as an effective nanoplatform for gene delivery and that their combination with DTX represents a promising strategy for the treatment of HCC.

## Supporting Information Available

Materials; polymer synthesis: techniques and equipment; glycopolymers’ characterization; physicochemical characterization of nanosystems; fluorescence microscopy of HepG2 cells transfected with PEG_45_-*b*-PAMA_168-_ and PEI-based polyplexes and untreated control cells; effect of the presence of asialofetuin on the transfection efficiency of HepG2 and Hep3B cells transfected with PEI-based polyplexes and untreated control; cellular internalization of PAMA-*co*-PLAMA-based polyplexes evaluated by flow cytometry; effect of endocytosis inhibitors on the transfection activity and toxicity of PAMA_114_-*co*-PLAMA_19_-and PEG_45_-*b*-PAMA_168_ based polyplexes; fluorescence microscopy and phase contrast of cells using fluorescein diacetate and PI staining for imaging live and dead cells; and cell viability evaluated by SRB assay (PDF)

## References

[ref1] Sung H.; Ferlay J.; Siegel R. L.; Laversanne M.; Soerjomataram I.; Jemal A.; Bray F. Global Cancer Statistics 2020: GLOBOCAN Estimates of Incidence and Mortality Worldwide for 36 Cancers in 185 Countries. Ca—Cancer J. Clin. 2021, 71, 209–249. 10.3322/caac.21660. 33538338

[ref2] Yang J. D.; Hainaut P.; Gores G. J.; Amadou A.; Plymoth A.; Roberts L. R. A Global View of Hepatocellular Carcinoma: Trends, Risk, Prevention and Management. Nat. Rev. Gastroenterol. Hepatol. 2019, 16, 589–604. 10.1038/s41575-019-0186-y. 31439937 PMC6813818

[ref3] Su T.-H.; Hsu S.-J.; Kao J.-H. Paradigm Shift in the Treatment Options of Hepatocellular Carcinoma. Liver Int. 2022, 42, 2067–2079. 10.1111/liv.15052. 34515412

[ref4] Llovet J. M.; Kelley R. K.; Villanueva A.; Singal A. G.; Pikarsky E.; Roayaie S.; Lencioni R.; Koike K.; Zucman-Rossi J.; Finn R. S. Hepatocellular Carcinoma. Nat. Rev. Dis. Prim. 2021, 7, 6 10.1038/s41572-020-00240-3. 33479224 PMC13537433

[ref5] Singh V.; Khan N.; Jayandharan G. R. Vector Engineering, Strategies and Targets in Cancer Gene Therapy. Cancer Gene Ther. 2022, 29, 402–417. 10.1038/s41417-021-00331-7. 33859378

[ref6] Gene Therapy Clinical Trials Worldwide. The Journal of Gene Medicine. https://a873679.fmphost.com/fmi/webd/GTCT (accessed August 11 2022).

[ref7] Sukumar U. K.; Rajendran J. C. B.; Gambhir S. S.; Massoud T. F.; Paulmurugan R. SP94-Targeted Triblock Copolymer Nanoparticle Delivers Thymidine Kinase-P53-Nitroreductase Triple Therapeutic Gene and Restores Anticancer Function against Hepatocellular Carcinoma in Vivo. ACS Appl. Mater. Interfaces 2020, 12, 11307–11319. 10.1021/acsami.9b20071. 32048820 PMC7997290

[ref8] Cordeiro R. A.; Mendonça P. V.; Coelho J.; Faneca H. Engineering Silica-Polymer Hybrid Nanosystems for Dual Drug and Gene Delivery. Biomater. Adv. 2022, 135, 212742 10.1016/j.bioadv.2022.212742. 35929215

[ref9] Malekshah O. M.; Chen X.; Nomani A.; Sarkar S.; Hatefi A. Enzyme/Prodrug Systems for Cancer Gene Therapy. Curr. Pharmacol. Rep. 2016, 2, 299–308. 10.1007/s40495-016-0073-y. 28042530 PMC5193473

[ref10] Vago R.; Collico V.; Zuppone S.; Prosperi D.; Colombo M. Nanoparticle-Mediated Delivery of Suicide Genes in Cancer Therapy. Pharmacol. Res. 2016, 111, 619–641. 10.1016/j.phrs.2016.07.007. 27436147

[ref11] Van Bruggen C.; Hexum J. K.; Tan Z.; Dalal R. J.; Reineke T. M. Nonviral Gene Delivery with Cationic Glycopolymers. Acc. Chem. Res. 2019, 52, 1347–1358. 10.1021/acs.accounts.8b00665. 30993967

[ref12] Ma Z.; Zhu X. X. Copolymers Containing Carbohydrates and Other Biomolecules: Design, Synthesis and Applications. J. Mater. Chem. B 2019, 7, 1361–1378. 10.1039/c8tb03162b. 32255007

[ref13] Pramudya I.; Chung H. Recent Progress of Glycopolymer Synthesis for Biomedical Applications. Biomater. Sci. 2019, 7, 4848–4872. 10.1039/c9bm01385g. 31650998

[ref14] Galbis J. A.; García-Martín M. D. G.; de Paz M. V.; Galbis E. Synthetic Polymers from Sugar-Based Monomers. Chem. Rev. 2016, 116, 1600–1636. 10.1021/acs.chemrev.5b00242. 26291239

[ref15] Thapa B.; Kumar P.; Zeng H.; Narain R. Asialoglycoprotein Receptor-Mediated Gene Delivery to Hepatocytes Using Galactosylated Polymers. Biomacromolecules 2015, 16, 3008–3020. 10.1021/acs.biomac.5b00906. 26258607

[ref16] Perrone F.; Craparo E. F.; Cemazar M.; Kamensek U.; Drago S. E.; Dapas B.; Scaggiante B.; Zanconati F.; Bonazza D.; Grassi M.; et al. Targeted Delivery of SiRNAs against Hepatocellular Carcinoma-Related Genes by a Galactosylated Polyaspartamide Copolymer. J. Controlled Release 2021, 330, 1132–1151. 10.1016/j.jconrel.2020.11.020. 33212117

[ref17] Lu J.; Wang J.; Ling D. Surface Engineering of Nanoparticles for Targeted Delivery to Hepatocellular Carcinoma. Small 2018, 14, 1702037 10.1002/smll.201702037. 29251419

[ref18] Ahmed M.; Narain R. The Effect of Molecular Weight, Compositions and Lectin Type on the Properties of Hyperbranched Glycopolymers as Non-Viral Gene Delivery Systems. Biomaterials 2012, 33, 3990–4001. 10.1016/j.biomaterials.2012.02.015. 22386601

[ref19] Dhande Y. K.; Wagh B. S.; Hall B. C.; Sprouse D.; Hackett P. B.; Reineke T. M. N-Acetylgalactosamine Block-Co-Polycations Form Stable Polyplexes with Plasmids and Promote Liver-Targeted Delivery. Biomacromolecules 2016, 17, 830–840. 10.1021/acs.biomac.5b01555. 26854615

[ref20] Smith A. E.; Sizovs A.; Grandinetti G.; Xue L.; Reineke T. M. Diblock Glycopolymers Promote Colloidal Stability of Polyplexes and Effective PDNA and SiRNA Delivery under Physiological Salt and Serum Conditions. Biomacromolecules 2011, 12, 3015–3022. 10.1021/bm200643c. 21657209

[ref21] Chen Y.; Diaz-Dussan D.; Peng Y.-Y.; Narain R. Hydroxyl-Rich PGMA-Based Cationic Glycopolymers for Intracellular SiRNA Delivery: Biocompatibility and Effect of Sugar Decoration Degree. Biomacromolecules 2019, 20, 2068–2074. 10.1021/acs.biomac.9b00274. 30970212

[ref22] Ahmed M.; Narain R. The Effect of Polymer Architecture, Composition, and Molecular Weight on the Properties of Glycopolymer-Based Non-Viral Gene Delivery Systems. Biomaterials 2011, 32, 5279–5290. 10.1016/j.biomaterials.2011.03.082. 21529936

[ref23] Peng Y. Y.; Diaz-Dussan D.; Kumar P.; Narain R. Tumor Microenvironment-Regulated Redox Responsive Cationic Galactose-Based Hyperbranched Polymers for SiRNA Delivery. Bioconjugate Chem. 2019, 30, 405–412. 10.1021/acs.bioconjchem.8b00785. 30525507

[ref24] Quan S.; Kumar P.; Narain R. Cationic Galactose-Conjugated Copolymers for Epidermal Growth Factor (EGFR) Knockdown in Cervical Adenocarcinoma. ACS Biomater. Sci. Eng. 2016, 2, 853–859. 10.1021/acsbiomaterials.6b00085. 33440481

[ref25] Ahmed M.; Deng Z.; Liu S.; Lafrenie R.; Kumar A.; Narain R. Cationic Glyconanoparticles: Their Complexation with DNA, Cellular Uptake, and Transfection Efficiencies. Bioconjugate Chem. 2009, 20, 2169–2176. 10.1021/bc900350c. 19919109

[ref26] Singhsa P.; Diaz-Dussan D.; Manuspiya H.; Narain R. Well-Defined Cationic N-[3-(Dimethylamino)Propyl]Methacrylamide Hydrochloride-Based (Co)Polymers for SiRNA Delivery. Biomacromolecules 2018, 19, 209–221. 10.1021/acs.biomac.7b01475. 29195038

[ref27] Peng Y. Y.; Diaz-Dussan D.; Kumar P.; Narain R. Acid Degradable Cationic Galactose-Based Hyperbranched Polymers as Nanotherapeutic Vehicles for Epidermal Growth Factor Receptor (EGFR) Knockdown in Cervical Carcinoma. Biomacromolecules 2018, 19, 4052–4058. 10.1021/acs.biomac.8b01066. 30157640

[ref28] Bockman M. R.; Dalal R. J.; Kumar R.; Reineke T. M. Facile Synthesis of GalNAc Monomers and Block Polycations for Hepatocyte Gene Delivery. Polym. Chem. 2021, 12, 4063–4071. 10.1039/d1py00250c.

[ref29] Li H.; Cortez M. A.; Phillips H. R.; Wu Y.; Reineke T. M. Poly(2-Deoxy-2-Methacrylamido Glucopyranose)-b -Poly(Methacrylate Amine)s: Optimization of Diblock Glycopolycations for Nucleic Acid Delivery. ACS Macro Lett. 2013, 2, 230–235. 10.1021/mz300660t. PMC3810285 24179703

[ref30] Chen J.; Wang K.; Wu J.; Tian H.; Chen X. Polycations for Gene Delivery: Dilemmas and Solutions. Bioconjugate Chem. 2019, 30, 338–349. 10.1021/acs.bioconjchem.8b00688. 30383373

[ref31] Herbst R. S.; Khuri F. R. Mode of Action of Docetaxel – a Basis for Combination with Novel Anticancer Agents. Cancer Treat. Rev. 2003, 29, 407–415. 10.1016/s0305-7372(03)00097-5. 12972359

[ref32] Wang L.; MacDonald R. C. Effects of Microtubule-Depolymerizing Agents on the Transfection of Cultured Vascular Smooth Muscle Cells: Enhanced Expression with Free Drug and Especially with Drug–Gene Lipoplexes. Mol. Ther. 2004, 9, 729–737. 10.1016/j.ymthe.2004.02.009. 15120334

[ref33] Vichai V.; Kirtikara K. Sulforhodamine B Colorimetric Assay for Cytotoxicity Screening. Nat. Protoc. 2006, 1, 1112–1116. 10.1038/nprot.2006.179. 17406391

[ref34] Alonso S. Exploiting the Bioengineering Versatility of Lactobionic Acid in Targeted Nanosystems and Biomaterials. J. Controlled Release 2018, 287, 216–234. 10.1016/j.jconrel.2018.08.030. 30149049

[ref35] Narain R.; Armes S. P. Synthesis and Aqueous Solution Properties of Novel Sugar Methacrylate-Based Homopolymers and Block Copolymers. Biomacromolecules 2003, 4, 1746–1758. 10.1021/bm034166e. 14606905

[ref36] Read E. S.; Thompson K. L.; Armes S. P. Synthesis of Well-Defined Primary Amine-Based Homopolymers and Block Copolymers and Their Michael Addition Reactions with Acrylates and Acrylamides. Polym. Chem. 2010, 1, 221–230. 10.1039/b9py00320g.

[ref37] Baker S. L.; Kaupbayeva B.; Lathwal S.; Das S. R.; Russell A. J.; Matyjaszewski K. Atom Transfer Radical Polymerization for Biorelated Hybrid Materials. Biomacromolecules 2019, 20, 4272–4298. 10.1021/acs.biomac.9b01271. 31738532

[ref38] Santo D.; Mendonça P. V.; Lima M. S.; Cordeiro R. A.; Cabanas L.; Serra A.; Coelho J. F. J.; Faneca H. Poly(Ethylene Glycol)-Block-Poly(2-Aminoethyl Methacrylate Hydrochloride)-Based Polyplexes as Serum-Tolerant Nanosystems for Enhanced Gene Delivery. Mol. Pharm. 2019, 16, 2129–2141. 10.1021/acs.molpharmaceut.9b00101. 30986077

[ref39] Bus T.; Traeger A.; Schubert U. S. The Great Escape: How Cationic Polyplexes Overcome the Endosomal Barrier. J. Mater. Chem. B 2018, 6, 6904–6918. 10.1039/c8tb00967h. 32254575

[ref40] Zhao L.; Li Y.; Pei D.; Huang Q.; Zhang H.; Yang Z.; Li F.; Shi T. Glycopolymers/PEI Complexes as Serum-Tolerant Vectors for Enhanced Gene Delivery to Hepatocytes. Carbohydr. Polym. 2019, 205, 167–175. 10.1016/j.carbpol.2018.10.036. 30446092

[ref41] Tan Z.; Dhande Y. K.; Reineke T. M. Cell Penetrating Polymers Containing Guanidinium Trigger Apoptosis in Human Hepatocellular Carcinoma Cells Unless Conjugated to a Targeting N-Acetyl-Galactosamine Block. Bioconjugate Chem. 2017, 28, 2985–2997. 10.1021/acs.bioconjchem.7b00598. 29193962

[ref42] Huang X.; Leroux J. C.; Castagner B. Well-Defined Multivalent Ligands for Hepatocytes Targeting via Asialoglycoprotein Receptor. Bioconjugate Chem. 2017, 28, 283–295. 10.1021/acs.bioconjchem.6b00651. 27966887

[ref43] Monnery B. D. Polycation-Mediated Transfection: Mechanisms of Internalization and Intracellular Trafficking. Biomacromolecules 2021, 22, 4060–4083. 10.1021/acs.biomac.1c00697. 34498457

[ref44] Kumar R.; Santa Chalarca C. F.; Bockman M. R.; Bruggen C. V.; Grimme C. J.; Dalal R. J.; Hanson M. G.; Hexum J. K.; Reineke T. M. Polymeric Delivery of Therapeutic Nucleic Acids. Chem. Rev. 2021, 121, 11527–11652. 10.1021/acs.chemrev.0c00997. 33939409

[ref45] Palermo E. F.; Lee D.-K.; Ramamoorthy A.; Kuroda K. Role of Cationic Group Structure in Membrane Binding and Disruption by Amphiphilic Copolymers. J. Phys. Chem. B 2011, 115, 366–375. 10.1021/jp1083357. 21171655 PMC3021096

[ref46] Becer C. R. The Glycopolymer Code: Synthesis of Glycopolymers and Multivalent Carbohydrate–Lectin Interactions. Macromol. Rapid Commun. 2012, 33, 742–752. 10.1002/marc.201200055. 22508520

[ref47] Ingle N. P.; Hexum J. K.; Reineke T. M. Polyplexes Are Endocytosed by and Trafficked within Filopodia. Biomacromolecules 2020, 21, 1379–1392. 10.1021/acs.biomac.9b01610. 32118406

[ref48] Hebbar M.; Ernst O.; Cattan S.; Dominguez S.; Oprea C.; Mathurin P.; Triboulet J. P.; Paris J. C.; Pruvot F. R. Phase II Trial of Docetaxel Therapy in Patients with Advanced Hepatocellular Carcinoma. Oncology 2006, 70, 154–158. 10.1159/000093007. 16645329

[ref49] Gu J.; Hao J.; Fang X.; Sha X. Factors Influencing the Transfection Efficiency and Cellular Uptake Mechanisms of Pluronic P123-Modified Polypropyleneimine/PDNA Polyplexes in Multidrug Resistant Breast Cancer Cells. Colloids Surf., B 2016, 140, 83–93. 10.1016/j.colsurfb.2015.12.023. 26741268

[ref50] Vaughan E. E.; Geiger R. C.; Miller A. M.; Loh-Marley P. L.; Suzuki T.; Miyata N.; Dean D. A. Microtubule Acetylation Through HDAC6 Inhibition Results in Increased Transfection Efficiency. Mol. Ther. 2008, 16, 1841–1847. 10.1038/mt.2008.190. 18781140

[ref51] Sheikh S.; Ernst D.; Keating A. Prodrugs and Prodrug-Activated Systems in Gene Therapy. Mol. Ther. 2021, 29, 1716–1728. 10.1016/j.ymthe.2021.04.006. 33831557 PMC8116605

[ref52] Vaughan H. J.; Zamboni C. G.; Hassan L. F.; Radant N. P.; Jacob D.; Mease R. C.; Minn I.; Tzeng S. Y.; Gabrielson K. L.; Bhardwaj P.; et al. Polymeric Nanoparticles for Dual-Targeted Theranostic Gene Delivery to Hepatocellular Carcinoma. Sci. Adv. 2022, 8, eabo6406 10.1126/sciadv.abo6406. 35857843 PMC9299552

[ref53] Faneca H.; Faustino A.; Pedroso de Lima M. C. Synergistic Antitumoral Effect of Vinblastine and HSV-Tk/GCV Gene Therapy Mediated by Albumin-Associated Cationic Liposomes. J. Controlled Release 2008, 126, 175–184. 10.1016/j.jconrel.2007.12.005. 18201792

[ref54] Barua S.; Rege K. The Influence of Mediators of Intracellular Trafficking on Transgene Expression Efficacy of Polymer–plasmid DNA Complexes. Biomaterials 2010, 31, 5894–5902. 10.1016/j.biomaterials.2010.04.007. 20452664

[ref55] Rodrigues T.; Kundu B.; Silva-Correia J.; Kundu S. C.; Oliveira J. M.; Reis R. L.; Correlo V. M. Emerging Tumor Spheroids Technologies for 3D in Vitro Cancer Modeling. Pharmacol. Ther. 2018, 184, 201–211. 10.1016/j.pharmthera.2017.10.018. 29097309

[ref56] Sant S.; Johnston P. A. The Production of 3D Tumor Spheroids for Cancer Drug Discovery. Drug Discovery Today: Technol. 2017, 23, 27–36. 10.1016/j.ddtec.2017.03.002. PMC5497458 28647083

