# Supplementary material for: Glycopolymers Mediate Suicide Gene Therapy in ASGPR-Expressing Hepatocellular Carcinoma Cells in Tandem with Docetaxel
Source: Biomacromolecules. 2023 Feb 13;24(3):1274–86. doi: 10.1021/acs.biomac.2c01329 (PMC10015461; doi:10.1021/acs.biomac.2c01329)
Supplement: Supplementary file 1 — bm2c01329_si_001.pdf [file bm2c01329_si_001.pdf]

Supporting Information

Glycopolymers mediate suicide gene therapy in ASGPR-expressing hepatocellular carcinoma cells in tandem with docetaxel

Daniela Santo [a,b], Rosemeyre A. Cordeiro[a,b], Patrícia V. Mendonça[c], Arménio C. Serra[c], Jorge F. J. Coelho[c,d], Henrique Faneca[a,b*]

[a] University of Coimbra, Center for Neuroscience and Cell Biology, Coimbra, Portugal

[b] University of Coimbra, Institute for Interdisciplinary Research, Coimbra, Portugal

[c] University of Coimbra, Centre for Mechanical Engineering, Materials and Processes, Department of Chemical Engineering, Coimbra, Portugal

[d]IPN - Instituto Pedro Nunes, Associação para a Inovação e Desenvolvimento em Ciência e Tecnologia, Rua Pedro Nunes, 3030-199 Coimbra, Portugal

Corresponding Author

*Henrique Faneca. Mailing address: Center for Neuroscience and Cell Biology, Edificio da Faculdade de Medicina, Universidade de Coimbra, Polo I, 1° piso Rua Larga, 3004- 504 Coimbra, Portugal. Tel.: +351-239-820-190. Fax: +351- 239-853-607. E-mail address: henrique@cnc.uc.pt.

Materials

2-Aminoethyl methacrylate hydrochloride (AMA; ≥95%, Polysciences), ascorbic acid (AscA; Sigma-Aldrich), ASGPR monoclonal antibody (Thermo Fisher), asialofetuin (Sigma-Aldrich), amiloride hydrochloride (Sigma-Aldrich), bovine serum albumin (BSA; Sigma-Aldrich), chlorpromazine (Sigma-Aldrich), copper(II) bromide (CuBr2; 99.999%, Sigma-Aldrich), deuterium oxide (D2O; Euroiso-top, +99.9% D), DC protein assay (Bio-Rad), 4,6-diamidino-2-phenylindole (DAPI, 1 μg mL−1) (Thermo Fisher Scientific), dimethylformamide (DMF, Fisher Scientific), docetaxel solution (DTX, HIKMA), Dulbecco's modified Eagle's medium-high glucose (DMEM-HG; Sigma-Aldrich), D-luciferin sodium salt (Synchem, 99%), ethyl α-bromophenyl acetate (EBPA; Alfa Aesar), filipin (Sigma-Aldrich), fluorescein o-methacrylate monomer (FMO, Sigma-Aldrich), fluorescein diacetate (Sigma-Aldrich) FluoroshieldTM (Sigma-Aldrich), Green Safe (Nzytech), lactobionic acid (Thermo Fisher Scientific), Lysotrack Red DND-99 (Thermo Fisher Scientific), PEI (branched, Mw 25 000) (Sigma-Aldrich), DNA plasmids encoding luciferase (pLuc), green fluorescent protein (GFP, pgfp) and HSV-TK (pTK) (Vical), resazurin sodium salt (Sigma-Aldrich), 2-propanol (Fisher Scientific), propidium iodide (Sigma-Aldrich). Methanol was dried over CaH2 and distilled before use. Triethylamine (≥99.5%, Sigma-Aldrich) was distilled before use. Tris(pyridine-2-ylmethyl)amine (TPMA) was synthesized as reported in the literature.[1]

Methods

Synthesis and characterization of glycopolymers

Techniques

A syringe pump (KDS Scientific, Legato 101) was used for the continuous feeding of the reducing agent at the rate of 1 μL/min. The molecular weight parameters of the polymers were determined by using a size exclusion chromatography (SEC) system equipped with an online degasser, a refractive index (RI) detector and a set of columns: Shodex OHpak SB-G guard column, OHpak SB-804HQ and OHpak SB-802.5HQ columns. The polymers were eluted at a flow rate of 0.5 mL/min with 0.1 M Na_2SO_4 (aq)/1 wt% acetic acid/0.02% NaN_3 at 40 °C. Before the injection, the samples were filtered through a polytetrafluoroethylene (PTFE) membrane with 0.45 μm pore. The system was calibrated with five narrow PEG standards and the polymers

number-average molecular weights (M_n^{SEC}) and dispersity ($Đ$ = M_w/M_n) were determined by conventional calibration using the Clarity software version 2.8.2.648.400. MHz ^{1}H NMR spectra were recorded on a Bruker Avance III 400 MHz spectrometer, with a 5-mm TIX triple resonance detection probe, in D_2O. Conversion of monomers was determined by integration of monomers NMR signals using the MestRenova software version: 10.0.1-14719.

Synthesis of 2-lactobionamidoethyl methacrylate (LAMA)

LAMA was synthesized according to a previously reported procedure.[2] Firstly, lactobionic acid was converted to the corresponding lactobionolactone. For that, lactobionic acid (4.0 g, 11.2 mmol) was dissolved in anhydrous methanol (25 mL) at 50 ºC, in the presence of trifluoroacetic acid as a catalyst (0.1 g, 1.1 mmol), followed by vacuum distillation to recover lactobionolactone. After that, lactobionolactone (1.5 g, 4.5 mmol) was dissolved in methanol at 50 ºC, followed by the addition of 2-aminoethyl methacrylate hydrochloride (1.5 g, 9.0 mmol), triethylamine (1.27 mL) and hydroquinone (0.05 g) at room temperature. The mixture was stirred for 6 h, concentrated by rotary evaporation and precipitated in 2-propanol. The white solid (LAMA) formed was filtered, washed with 2-propanol and dried under vacuum (yield = 81%). Then, their chemical structure was characterized by 1H NMR spectroscopy (Figure 1a and Figure S2a, Supporting Information) and SEC (Figure 1b and Figure S2b, Supporting Information).

Synthesis of fluorescein-labeled glycopolymer

AMA (0.54g, 3.7 mmol), LAMA (0.25 g, 532 µmol), fluorescein o-methacrylate (FMO) (53.3 mg, 133 µmol), $CuBr_2$ (2.97 mg, 13.3 µmol), TPMA (15.5 mg, 53 µmol), and EBPA (6.5 mg, 26 µmol) were dissolved in water/DMF mixture (50/50, V/V) (3.5mL). The mixture was added to a 10 mL round-bottom Schlenk flask, equipped with a magnetic stir bar, and purged with nitrogen for 30 min. The flask was placed in an oil bath at 60 °C and a deoxygenated AscA solution (43 mM) was continuously injected into the reaction medium using a syringe pump at the rate of 1 µL/min. The reaction was stopped after 3 h, and a sample was collected for ^{1}H NMR spectroscopy to determine the monomers (FMO, AMA and LAMA) conversion. The final reaction mixture was dialyzed (dialysis membrane MWCO = 3500) against deionized water and the glycopolymer was recovered by freeze-drying.

Physicochemical characterization of polyplexes

Green Safe Intercalation Assay

The accessibility to DNA of the polyplexes was analyzed using Green Safe intercalation assay. The polyplexes were prepared as described above and after 15 min, 50 µL of each sample was transferred into a black 96-well plate (Costar, Cambridge, CA, USA). Then, 50 µL of Green Safe solution (0.00002 % (V/V)) was added to polyplexes. Following 10 min incubation, fluorescence was measured in a SpectraMax Gemini EM fluorometer (Molecular Devices, Sunnyvale, CA, USA) at the excitation wavelength of 490 nm and emission wavelength of 530 nm. The fluorescence scale was calibrated such that the initial fluorescence of Green Safe (50 µL of Green Safe solution was added to 50 µL of Milli-Q water) was set as residual fluorescence. The value of fluorescence obtained with 1 µg of naked DNA (control) was set as 100%. The amount of DNA available to interact with the probe was calculated by subtracting the values of residual fluorescence from those obtained for the samples and expressed as the percentage of the control.

Agarose Gel Electrophoresis Assay

To evaluate the complexation of the DNA with the copolymers an electrophoresis in agarose gel was performed. Polyplexes were prepared and, after 15 min, 20 µL of each sample was added to 5 µL of loading buffer. 20 µL of each blend were transferred to a 1% agarose gel prepared in TBE solution and containing 1.5 µL of Green Safe. The electrophoresis was set to 30 min at 80 mV. Sample visualization takes place in a GelDoc® (BioRad®, USA) system using the QuantityOne® program.

Dynamic Light Scattering and Zeta Potential Analysis

Dynamic light scattering (DLS) measurements were performed on a Zetasizer Nano-ZS (Malvern Instruments Ltd. UK). The particle size distribution (in intensity) and average hydrodynamic diameter (z-average) were determined with Zetasizer 7.13 software. Measurements were made at 25 °C and at a backward scattering angle of 173°. ζ-Potential measurements were performed using a Zetasizer Nano-ZS (Malvern Instruments Ltd., UK) coupled to laser Doppler electrophoresis and determined using a Smoluchovski model.

Cell Culture

Human hepatocellular carcinoma cells (HepG2 and Hep3B cell lines) and human epithelial cervical carcinoma cells (HeLa cell line) were maintained at 37 °C, under 5% CO_2, in Dulbecco's modified Eagle's medium-high glucose (DMEM-HG, Sigma-Aldrich), supplemented with 10% (V/V) heat inactivated fetal bovine serum (FBS, Sigma-Aldrich), penicillin (100 U/mL), and streptomycin (100 μg/mL). All cells were grown in monolayers and were detached by treatment with a 0.25% trypsin solution (Sigma-Aldrich).

Transfection Activity

The biological activity of the different polyplexes was determined by luminescence, using luciferase as a reporter gene (pLuc plasmid), in HepG2 and Hep3B cells. Briefly, the HepG2 (8×10^4 cells/well), Hep3B (3.5×10^4 cells/well) and HeLa (5×10^4 cells/well) cells were seeded onto 48-well culture plates 24 h prior to incubation with polyplexes. The cells were used at 70% confluence, and polyplexes containing 1 μg of p.Luc were added to the cells previously covered with DMEM-HG containing 10% (v/v) FBS. After 4 h of incubation, the transfection medium was replaced with fresh DMEM-HG, and the cells were further incubated for 48 h. At this time, the cells were washed twice with PBS, and 100 μL of lysis buffer was added to each well. The quantification of luciferase expression in cell lysates was evaluated by measuring the light production by luciferase in a FLUOstar Omega Microplate Reader (BMG Labtech, USA). The protein content of the lysates was measured by the DC protein assay reagent (Biorad, CA, USA) using BSA as a standard. The data were expressed as relative light units of luciferase per milligram of total cell protein. For the competitive studies, the culture medium containing 1 mg/mL of asialofetuin (ASF) or 40 μg/ml of antibody against the ASGP-R (ASGPR1 Ab) was added to cells 1 h before the addition of nanosystems and maintained during the 4 h of transfection. In the endocytic pathway studies, the culture medium containing different inhibitors (75 μM chlorpromazine, 0.25 mM amiloride or 2 μg/mL filipin) was added to cells 1 h before the polyplex addition and maintained during the 4 h of transfection. To evaluate the effect of docetaxel (DTX) as enhancer of transfection activity, the culture medium containing different concentrations of DTX (0.003; 0.006; 0.0125; 0.025; 0,05 or 0.1 μM) was added to cells 1 h before the addition of nanosystems and maintained during the 4 h of transfection.

Transfection Efficiency

To evaluate the transfection efficiency of our formulations, green fluorescent protein (GFP) expression was evaluated in HCC cells by fluorescence microscopy. Briefly, the HepG2 (1.1×10^5 cells/well) and Hep3B (7.5×10^4 cells/well) cells were seeded on 24-well plates (the wells were previously covered with a coverslip), and after 24 h, polyplexes containing 2 μg of pgfp were added to the cells previously covered with 0.5 mL of DMEM-HG with serum. After 4 h of incubation (5% CO_2 at 37 °C), the transfection medium was replaced with DMEM-HG containing 10% (V/V) FBS and antibiotics, and the cells were further incubated for 48 h. After that, the cells were washed twice with PBS, fixed with 4% paraformaldehyde for 15 min at room temperature, and then mounted in Fluoroshield medium. The images (original magnification ×20) were obtained on an Axio Imager Z2 microscope (Zeiss, Munich, Germany) using an AxioCam HRc camera (Zeiss, Germany).

Cell Viability Assay

Cell viability under different experimental conditions was assessed by an Alamar Blue assay. After 48 h of transfection, the cells were incubated with DMEM containing 10% (V/V) Alamar Blue dye, prepared from a 0.1 $mg.mL^{-1}$ stock solution of Alamar Blue. After 1 h incubation period at 37 °C, the absorbance of the medium was measured at 570 (A_{570}) and 600 (A_{600}) nm in SPECTRAmax PLUS 384 spectrophotometer (Molecular Devices, USA). Cell viability was calculated, as percentage of the nontreated control cells, according to the formula: $[(A_{570} - A_{600})$ of treated cells $\times 100]/[(A_{570} - A_{600})$ of control cells].

Cell uptake

Polyplexes were prepared with 1% of fluorescein-labeled FMO_2-*co*-$PAMA_{103}$-*co*-$PLAMA_{19}$ at their optimal N/P ratio. HepG2 cells were seeded on 24-well plates at a density of 1.6×10^5 cells/well and, after 24 h, polyplexes containing the glycopolymer labeled with fluorescein were added to cells previously covered with 0.5 mL of DMEM-HG with serum. After 4 h incubation (5% CO_2 at 37 °C), cells were washed twice with PBS, detached with trypsin, and then washed and resuspended in PBS. To quench external fluorescence, trypan blue was added to each sample 1 min before FACSCalibur flow cytometer (Becton Dickinson, NJ, USA) analysis, at a final concentration of 0.05% (V/V). In the competitive studies, the culture medium containing 2

mg/mL of asialofetuin was added to cells 30 min before the addition of nanosystems and maintained during the 4 h of transfection. Live cells were gated by forward/side scattering from a total of 20000 events, and data was analyzed using FlowJo software.

Intracellular distribution of polyplexes

Confocal laser scanning microscopy was used to visualize the intracellular distribution of polyplexes prepared with 1% of fluorescein-labeled glycopolymer at their optimal N/P ratio. The HepG2 cells were seeded in 24-well culture plates (previously covered with a coverslip) at an initial density of 1.5×10^5 cells/well and, after 24 h, polyplexes were added to the cells previously covered with 0.5 mL of DMEM-HG with serum. In the competitive studies, the culture medium containing 2 mg/mL of asialofetuin was added to cells 30 min before the addition of nanosystems and maintained during the 4 h of transfection. After this period of incubation (5% CO_2 at 37 °C), the transfection medium was removed, and the cells were washed with PBS and incubated for 30 min with 200 nM Lysotrack Red DND-99, which labels the acidic compartments of living cells. Thereafter, the cells were washed three times with PBS and fixed with 4% paraformaldehyde solution for 15 min at room temperature. Nuclei labeling was accomplished through 5 min of incubation at room temperature with the fluorescent DNA binding dye DAPI (1 μg/mL). The cells were then mounted in Fluoroshield medium, and images were taken in a Zeiss LSM 710 Axio Observer microscope (Zeiss, Gottingen, Germany) with a Plan-Apochromat 63×/1.40 oil differential interference contrast (DIC) M27 objective at the excitation wavelengths of 405 nm for DAPI (blue), 488 nm for fluorescein (green), and 561 nm for Lysotracker (red).

Antitumor activity

To evaluate the antitumor effect of the developed therapeutic strategy in a 3D cell culture model, 3×10^3 $HepG^2$ cells/well were placed in 96-well cell culture round-bottom ultralow attachment microplates. After the initial 3 days of formation, spheroids were incubated, in the presence or absence of DTX (0.006 μM), with $PAMA_{144}$-*co*-$PLAMA_{19}$-based polyplexes, prepared at 50/1

N/P ratio with 0.5 μg of pTK per well. After 24 h of incubation, the cell culture medium was renewed by culture medium with or without GCV (100 μM) every 48 h. Microscopy images were obtained at 72 h, 120 h and 168 h. The images were acquired with x20 magnification (planapochromat objectives) in an Axio Observer Z1 widefield microscope coupled to digital CMOS camera (ORCA Flash 4.0) (Zeiss®,Germany) and analyzed with Zen Blue software (Zeiss®, Germany). Analysis of spheroid areas was performed using the Zen Blue software. Fluorescence images were obtained using fluorescein diacetate (5 mg/mL in acetone) and PI (5 mg/mL in PBS) for live/dead staining of spheroids. After 45 min of incubation, the spheroids were washed and observed immediately. The PI mean fluorescence intensity (MFI) per spheroid area was calculated using ImageJ software. Six spheroids per treatment condition were analyzed and the results are representative of three independent experiments.

Results

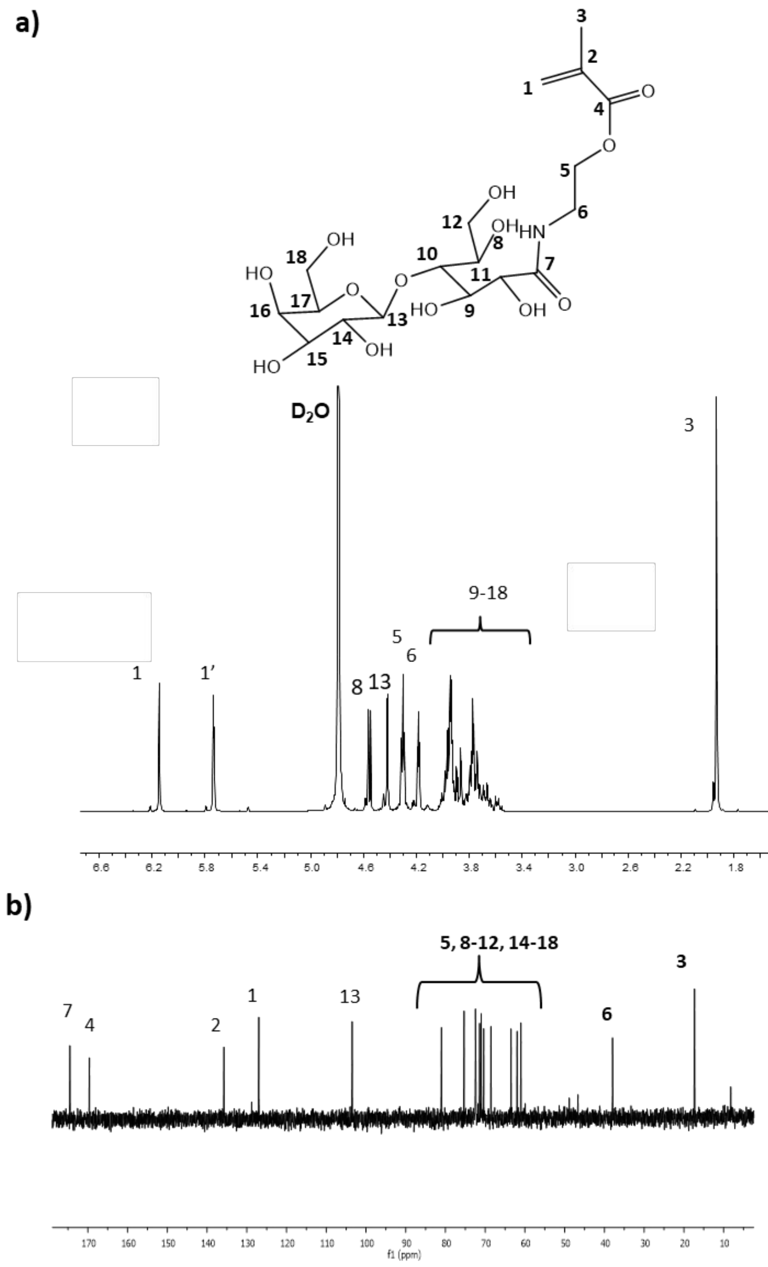

Figure S1 – ^{1}H (a) and ^{13}C (b) NMR spectra (D_2O, 400 MHz) for the LAMA monomer.

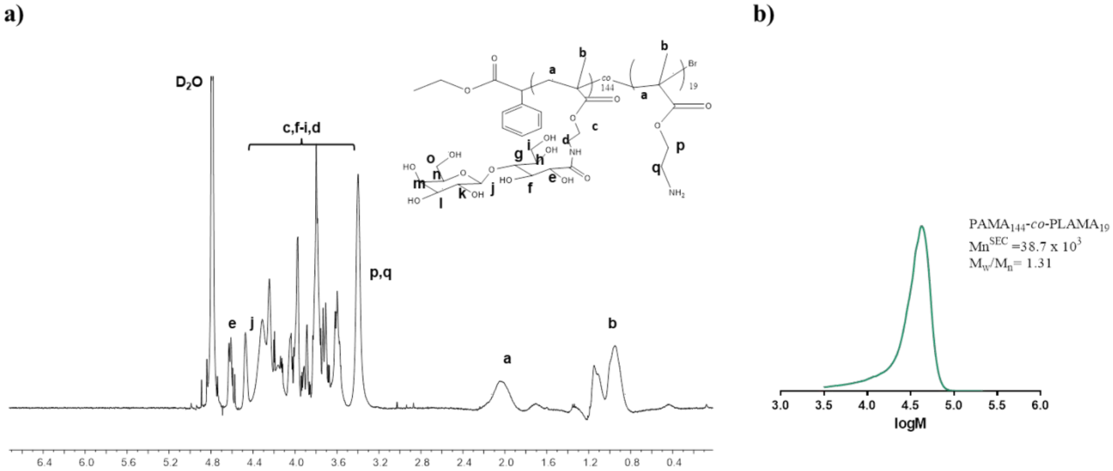

Figure S2 - ^{1}H NMR spectrum (D_2O, 400 MHz) (a) and SEC trace (b) of the $PAMA_{144}$-*co*-$PLAMA_{19}$ random copolymer prepared by ARGET ATRP.

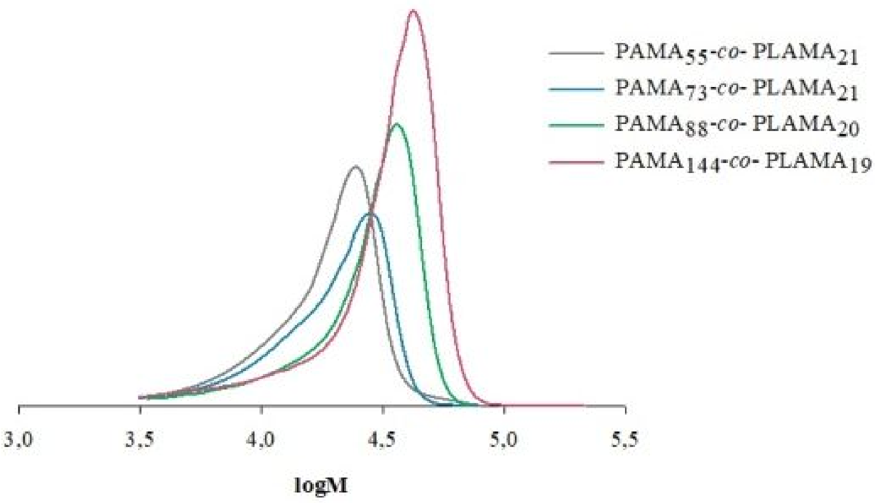

Figure S3 - SEC traces of the PAMA-*co*-PLAMA random copolymers prepared by ARGET ATRP.

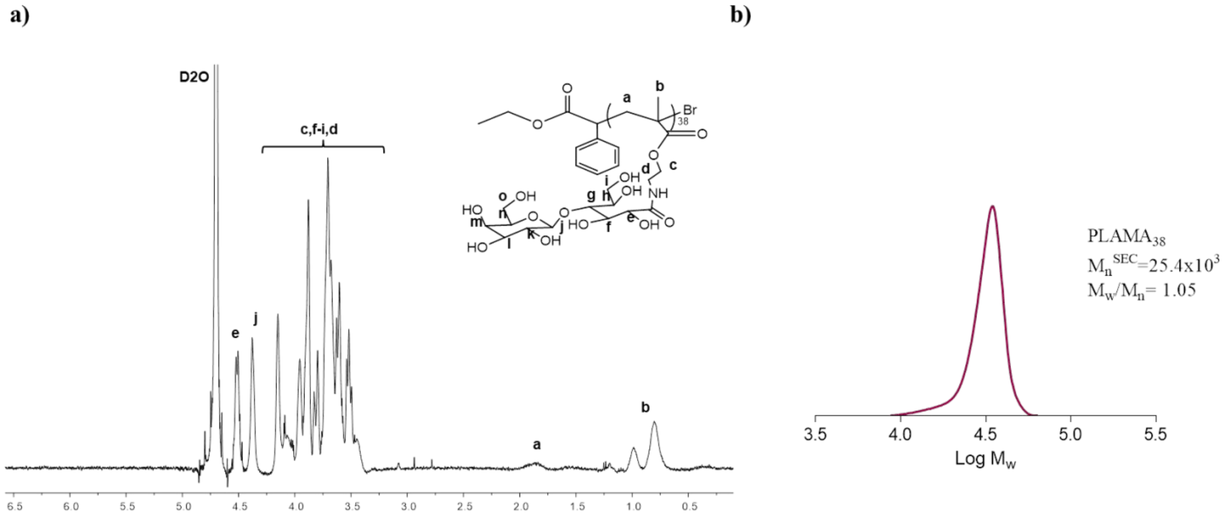

Figure S4 – ^{1}H NMR spectrum (D_2O, 400 MHz) (a) and SEC trace (b) of the $PLAMA_{38}$-Br homopolymer prepared by ARGET ATRP.

Acid-base Titration for pKa Determination

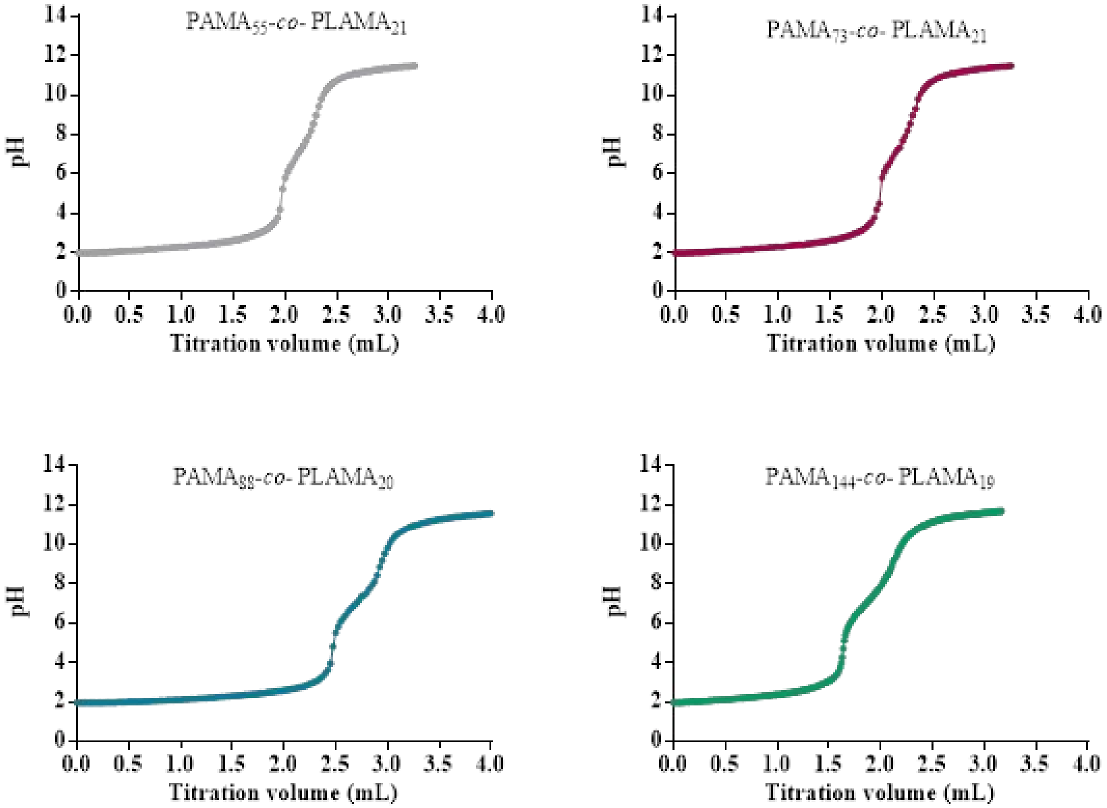

Glycopolymer	*pKa*
PAMA$_{55}$-*co*- PLAMA$_{21}$	7.2
PAMA$_{73}$-*co*- PLAMA$_{21}$	7.2
PAMA$_{88}$-*co*- PLAMA$_{20}$	7.1
PAMA$_{144}$-*co*- PLAMA$_{19}$	7.0

Figure S5 - Potentiometric titration curves of PAMA-co-PLAMA glycopolymers. Glycopolymers were dissolved in acidic water (pH=3) and the polymeric solutions were acidified to pH 2 with 1% (V/V) HCl and titrated with 0.1M NaOH.

Physicochemical Characterization of Nanosystems

The physicochemical characteristics of polymeric-based nanocarriers play an important role in their capacity to mediate gene delivery into target cells. These include the ability to condense and protect DNA, the size, and the surface charge of the developed polyplexes. Thus, the physicochemical properties of PAMA-*co*-PLAMA-based nanocarriers were determined to evaluate their influence on transfection activity (Figure S6).

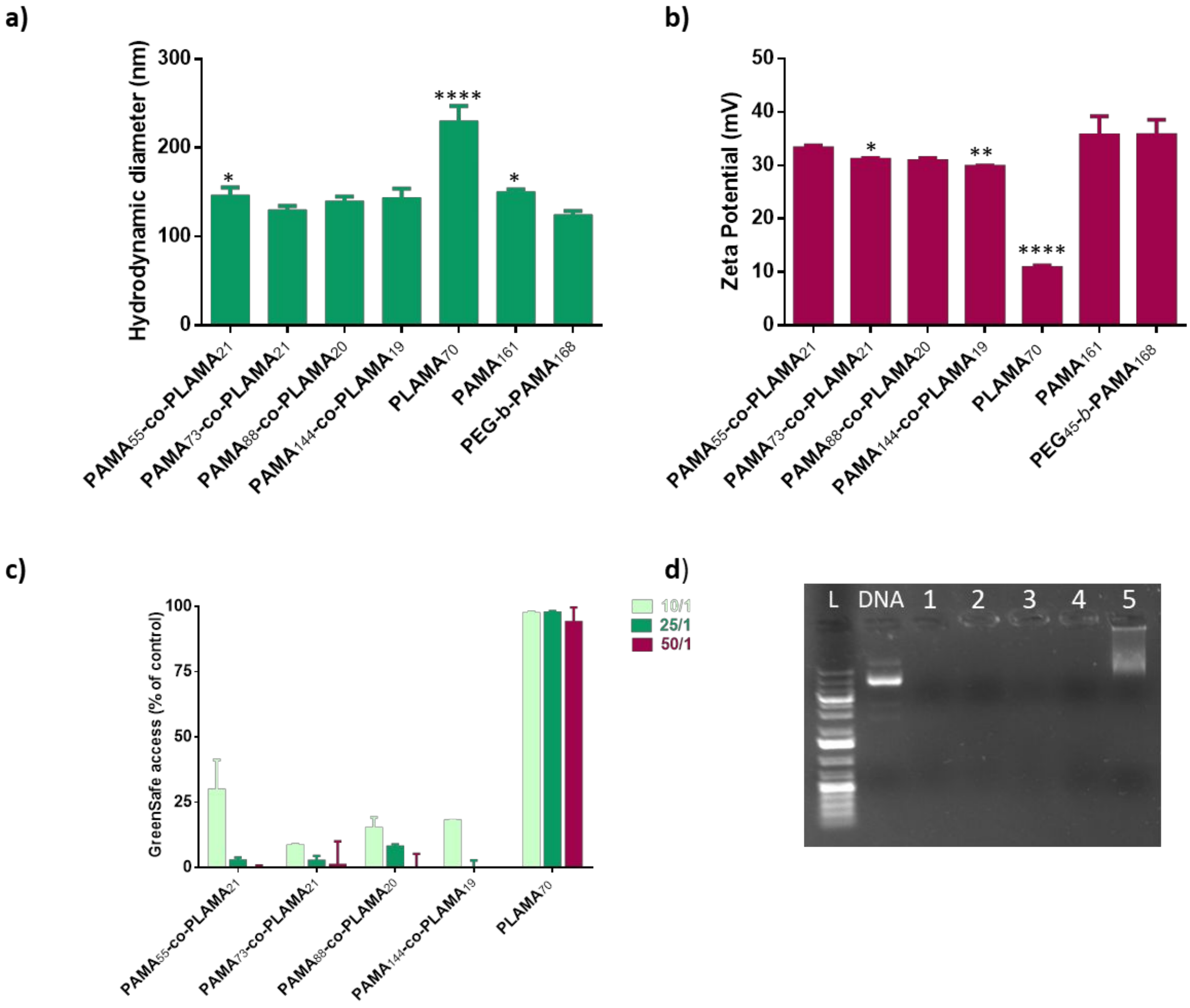

Figure S6 – Physicochemical characterization of PAMA-co-PLAMA-based polyplexes. (a) Hydrodynamic diameter and (b) zeta potential of PAMA$_{55}$-*co*-PLAMA$_{21}$-, PAMA$_{73}$-*co*-PLAMA$_{21}$-, PAMA$_{88}$-*co*-PLAMA$_{20}$-, PLAMA$_{70}$-, PAMA$_{161}$- and PEG$_{45}$-*b*-PAMA$_{168}$-based polyplexes prepared at 50:1 N/P ratio, and PAMA$_{144}$-*co*-PLAMA$_{19}$- based polyplexes formulated at 25:1 N/P ratio. Asterisks (****$p < 0.0001$, **$p < 0.01$, and *$p < 0.05$) indicate values with statistical significance when compared to those obtained with PEG$_{45}$-*b*-PAMA$_{168}$-based polyplexes. (c) DNA complexation efficiency. (d) Gel electrophoresis of polyplexes prepared with different glycopolymers: L- DNA ladder (1 kb plus), DNA - Plasmid only, 1 - PAMA$_{55}$-*co*-PLAMA$_{21}$, 2 - PAMA$_{73}$-*co*-PLAMA$_{21}$, 3 - PAMA$_{88}$-*co*-PLAMA$_{20}$, 4 - PAMA$_{144}$-*co*-PLAMA$_{19}$, 5 - PLAMA$_{70}$.

The DLS measurements, presented in Figure S6a, revealed that all PAMA-*co*-PLAMA glycopolymers were able to efficiently condense pDNA into nanosized (130–150 nm) polyplexes. In addition, the results showed that the nanocarriers prepared with glycopolymers with different cationic contents presented similar hydrodynamic diameter, which may be justified by the fact that at the tested N/P ratios, a maximum condensation of genetic material was reached

and, consequently, no significant changes in size were observed. Moreover, $PLAMA_{70}$ homopolymer generated larger nanoparticles than cationic-containing glycopolymers, suggesting that they have a limited ability to condense genetic material.

Regarding the zeta potential of the developed nanocarriers, the results illustrated in Figure 2b, showed that their surface charge is positive, ranging between +10 and +35 mV. In addition, the results revealed that all the developed PAMA-*co*-PLAMA-based nanocarriers exhibited similar surface charge, which was slightly lower than that obtained for $PAMA_{161}$- and PEG_{45}-*b*-$PAMA_{168}$-based polyplexes. Moreover, nanocarriers prepared with the carbohydrate homopolymer exhibited lower zeta potential than polyplexes prepared with cationic-containing copolymers. These results indicate that carbohydrate-based nanosystems, when compared to PEGylated nanovehicles, provide a superior hydrophilic hindrance, which improves their capability to mask the excess of positive charge of nanocarriers.

To evaluate if the developed nanocarriers were able to condense the plasmid DNA, the Green Safe intercalation assay was performed. The results showed a decrease of Green Safe fluorescence with the increase of the N/P ratio of glycoplexes for all the developed formulations, except for $PLAMA_{70}$-based nanocarriers (Figure S6c). The glycoplexes generated with PLAMA

homopolymer had the highest levels of intercalating agent access. This fact can be explained by the nature of the interactions of this glycopolymer with DNA, which probably occurs mostly via hydrogen bonding. On the other hand, for all developed PAMA-*co*-PLAMA-based polyplexes prepared at 25/1 and 50/1 N/P ratios, the results showed that these nanocarriers provided almost complete DNA condensation and protection.

The data obtained in the Green safe accessibility assays are consistent with those obtained in the agarose gel electrophoresis assays (Figure S6d).

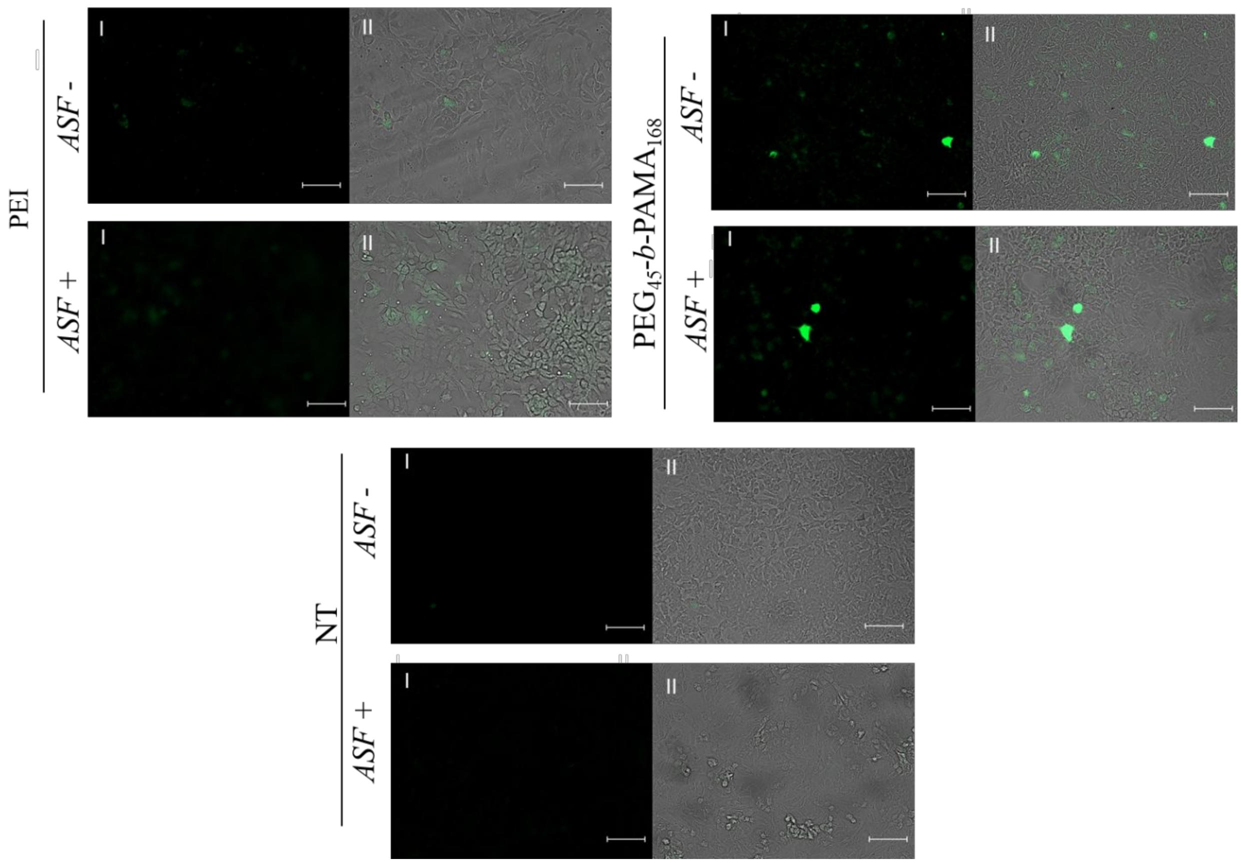

Figure S7– Effect of the presence of asialofetuin on the transfection efficiency of PEI- and PEG_{45}-*b*-$PAMA_{168}$ based polyplexes in HepG2 cells. Typical fluorescence images (I) and overlapping of fluorescence microscopy and phase contrast images (II) of cells (scale bar = 10 μm).

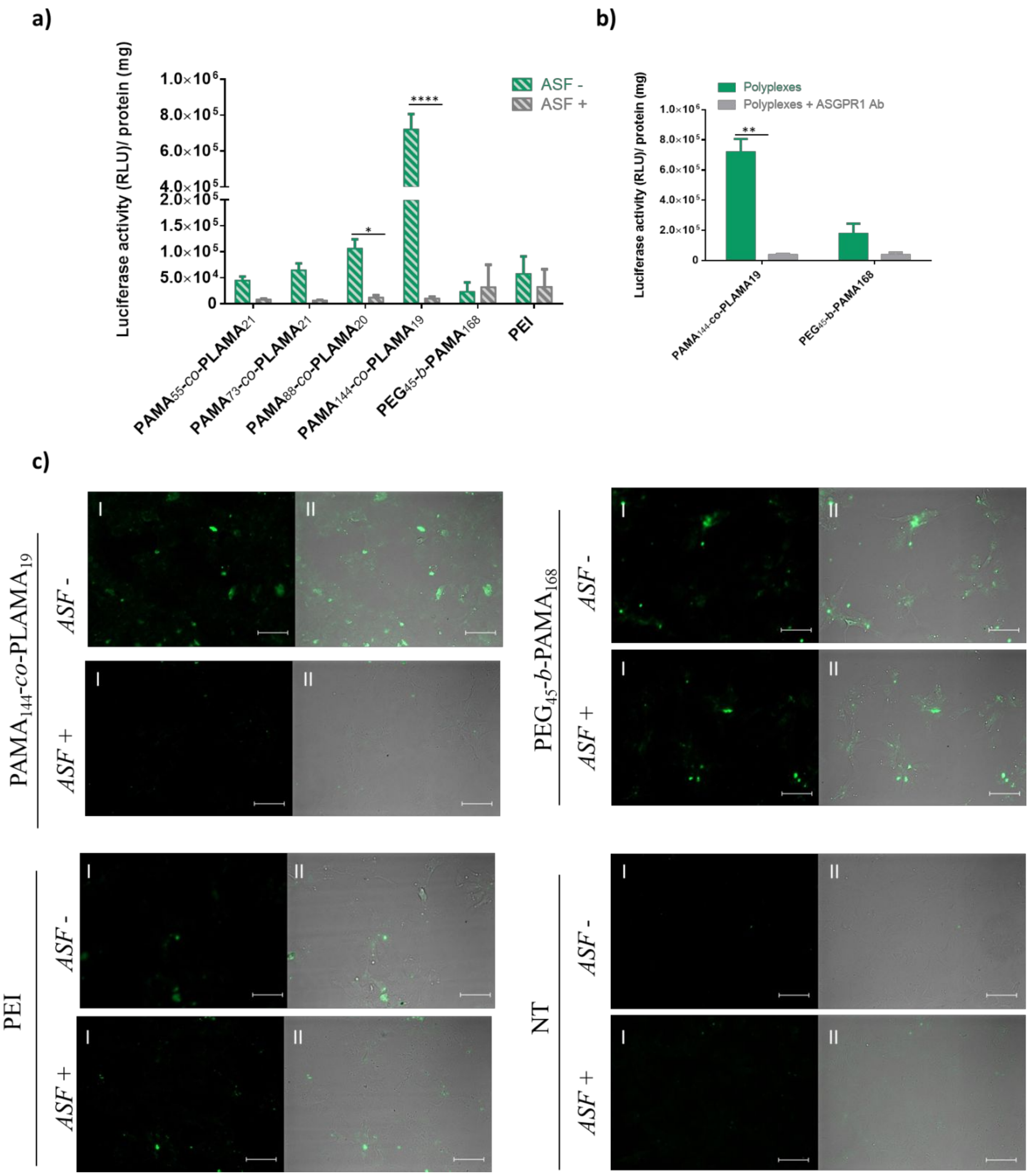

Figure S8 – Effect of the presence of asialofetuin and an antibody against the ASGPR on biological activity of PAMA-*co*-PLAMA-based polyplexes in Hep3B cells. (a, b) Asterisks (****p < 0.0001, **p < 0.01 and *p < 0.05) correspond to values that differ significantly from those obtained with the same nanocarrier in the absence of ASGPR-competition agent. (c) Typical fluorescence images (I) and overlapping of fluorescence microscopy and phase contrast images (II) of cells transfected with different nanocarriers and non-treated cells in the presence and absence of asialofetuin (scale bar = 50 μm). Typical fluorescence images (I) and

overlapping (II) of fluorescence microscopy and phase contrast images of cells transfected with different glycopolymers-based nanocarriers (scale bar = 50 µm).

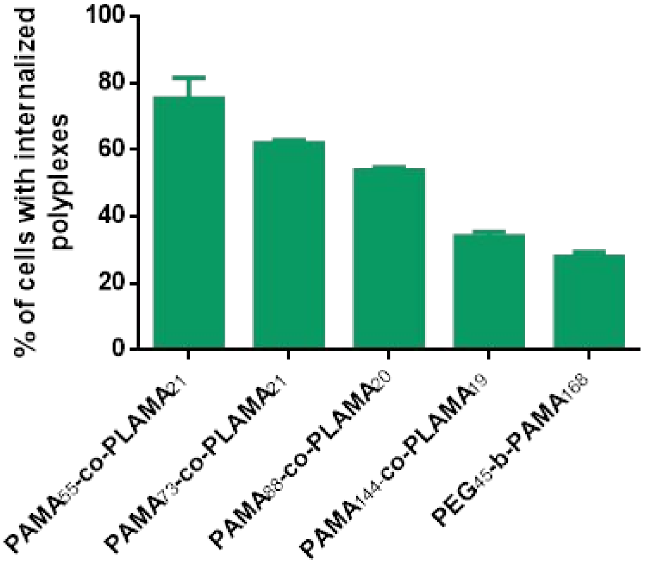

Figure S9 – Cellular internalization of PAMA-*co*-PLAMA-based polyplexes evaluated by flow cytometry. Polyplexes were prepared by complexing the copolymers, containing 1% fluorescein-labeled glycopolymer, with 2 µg of DNA plasmid encoding luciferase at their optimal N/P ratios.

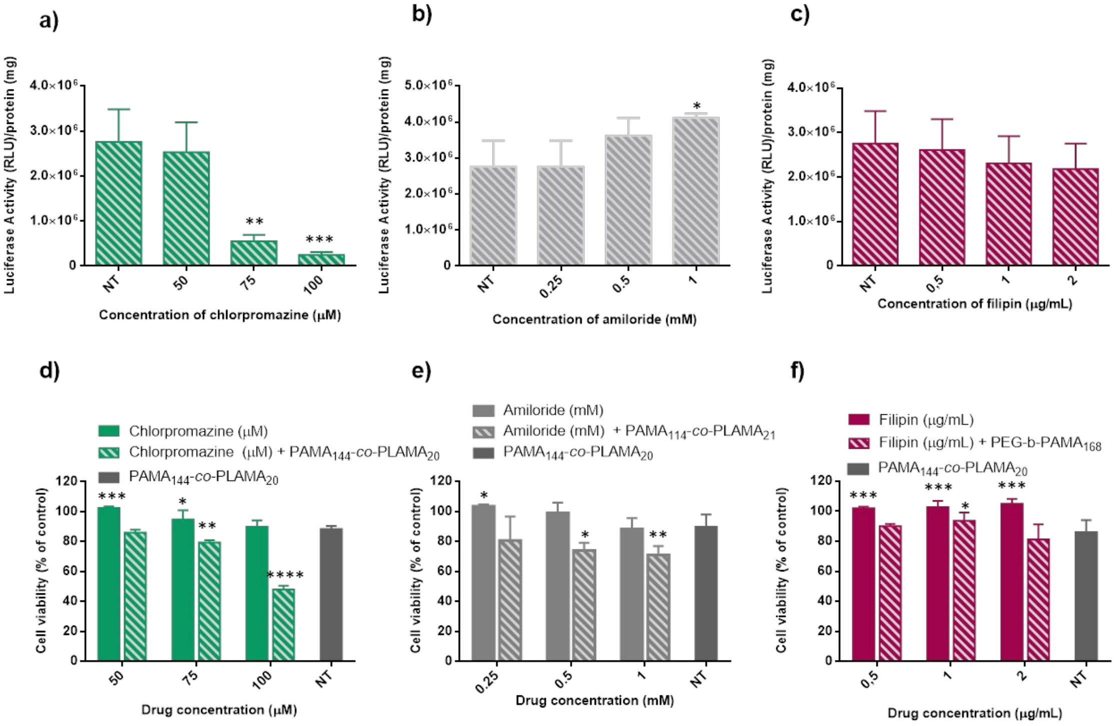

Figure S10 – Effect of endocytosis inhibitors on the transfection activity (a), (b), (c) and toxicity (d), (e), (f) of $PAMA_{114}$-*co*-$PLAMA_{21}$-based polyplexes. HepG2 cells were pretreated with chlorpromazine (50; 75; 100 µM) or amiloride (0.25; 0.5; 1 mM) or filipin (0.5; 1; 2 µg.mL^{-1}) and transfected with $PAMA_{114}$-*co*-$PLAMA_{21}$-based polyplexes prepared with 1 µg of pCMV.Luc at 25/1 N/P ratio. Asterisks (****p <0.0001, ***p < 0.001, **p < 0.01 and *p < 0.05) indicate values that differ significantly from those measured in the control (cells not-treated with endocytosis inhibitors).

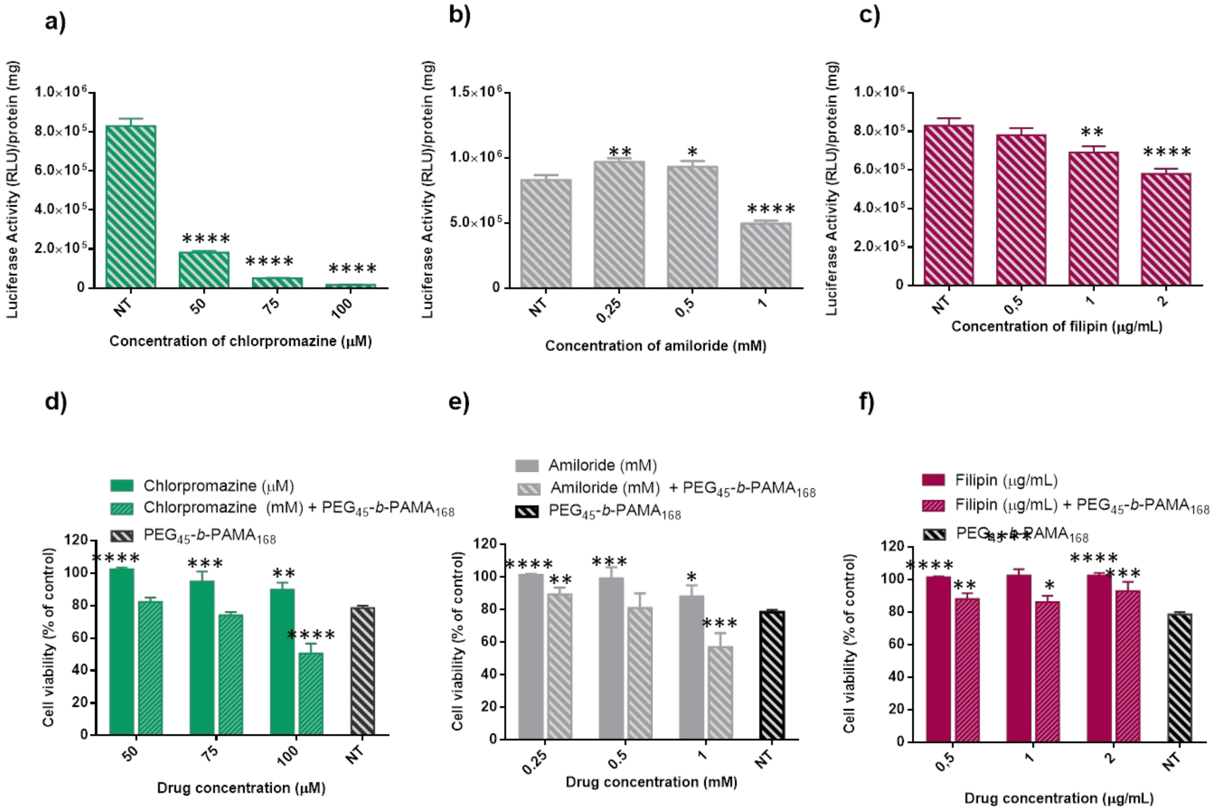

Figure S11 – Effect of endocytosis inhibitors on the transfection activity (a), (b), (c) and toxicity (d), (e), (f) of PEG45-b-PAMA168-based polyplexes. HepG2 cells were pretreated with chlorpromazine (50; 75; 100 µM) or amiloride (0.25; 0.5; 1 mM) or filipin (0.5; 1; 2 µg.mL-1) and transfected with PEG45-b-PAMA168-based polyplexes prepared with 1 µg of pCMV.Luc at 50/1 N/P ratio. Asterisks (****p <0.0001, ***p < 0.001, **p < 0.01 and *p < 0.05) indicate values that differ significantly from those measured in the control (cells not-treated with endocytosis inhibitors).

a)

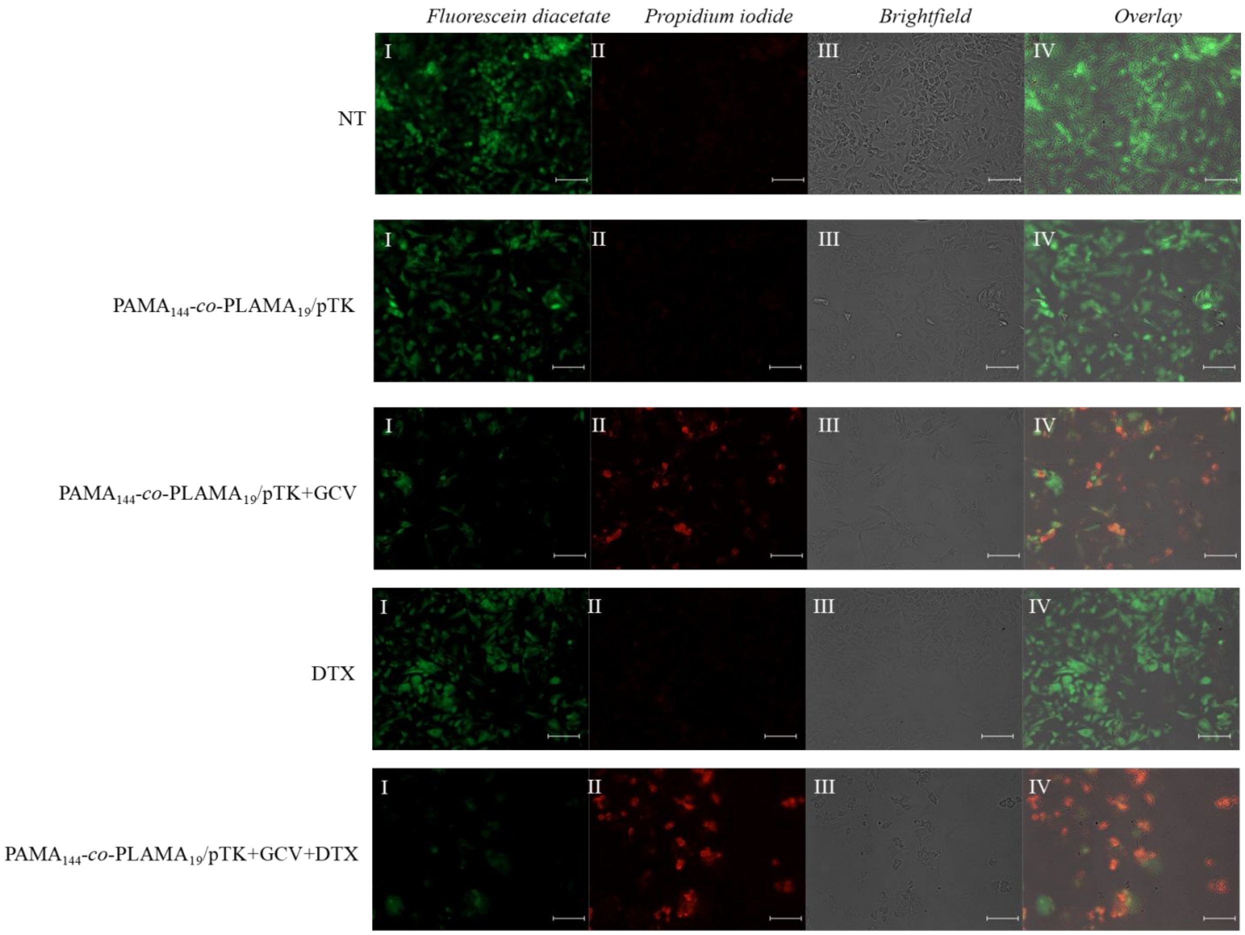

b)

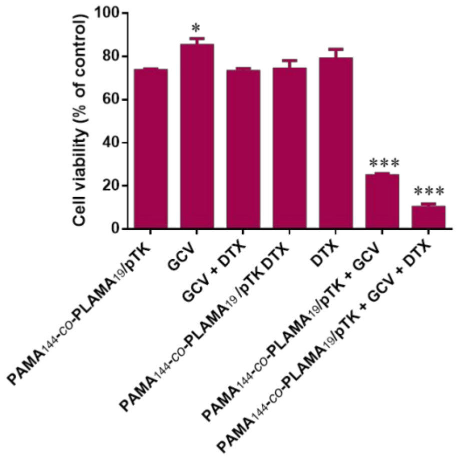

Figure S12 – Therapeutic potential of the suicide gene therapy strategy mediated by the glycopolymer-based nanocarrier combined with docetaxel in HepG2 cells. a) Representative images of fluorescence microscopy and phase contrast of cells using fluorescein diacetate (green)

and propidium iodide (red) staining for imaging live and dead cells, respectively (scale bar = 50 µm). b) Cell viability evaluated by the SRB assay. Asterisks (****p <0.0001, □□□p<0.001) indicate values that significantly differ from those measured for cells transfected with PAMA144-co-PLAMA19-based nanocarriers, containing 1 µg of pTK plasmid.

References

(1) Britovsek, G. J. P.; England, J.; White, A. J. P. Non-Heme Iron(II) Complexes Containing Tripodal Tetradentate Nitrogen Ligands and Their Application in Alkane Oxidation Catalysis. *Inorg. Chem.* **2005**, *44* (22), 8125–8134.

(2) Narain, R.; Armes, S. P. Synthesis and Aqueous Solution Properties of Novel Sugar Methacrylate-Based Homopolymers and Block Copolymers. *Biomacromolecules* **2003**, *4* (6), 1746–1758.
